# Synthesis and pharmacological evaluation of novel isoquinoline *N*-sulphonylhydrazones designed as ROCK inhibitors

**DOI:** 10.1080/14756366.2018.1490732

**Published:** 2018-07-25

**Authors:** Ramon Guerra de Oliveira, Fabiana Sélos Guerra, Cláudia dos Santos Mermelstein, Patrícia Dias Fernandes, Isadora Tairinne de Sena Bastos, Fanny Nascimento Costa, Regina Cely Rodrigues Barroso, Fabio Furlan Ferreira, Carlos Alberto Manssour Fraga

**Affiliations:** a Laboratório de Avaliação e Síntese de Substâncias Bioativas (LASSBio^®^), Instituto de Ciências Biomédicas, Universidade Federal do Rio de Janeiro, Rio de Janeiro, Brazil;; b Programa de Pós-Graduação em Farmacologia e Química Medicinal, Instituto de Ciências Biomédicas, Universidade Federal do Rio de Janeiro, Rio de Janeiro, Brazil;; c Laboratório de Farmacologia da Dor e da Inflamação, Instituto de Ciências Biomédicas, Universidade Federal do Rio de Janeiro, Rio de Janeiro, Brazil;; d Laboratório de Diferenciação Muscular, Instituto de Ciências Biomédicas, Universidade Federal do Rio de Janeiro, Rio de Janeiro, Brazil;; e Instituto de Física, Universidade do Estado do Rio de Janeiro, Rio de Janeiro, Brazil;; f Centro de Ciências Naturais e Humanas (CCNH), Universidade Federal do ABC (UFABC), São Paulo, Brazil

**Keywords:** Molecular hybridization, *N*-sulphonylhydrazone, Rho kinase, ROCK inhibitor, fasudil, LASSBio-1524

## Abstract

In this study, we synthesized a new congener series of *N*-sulphonylhydrazones designed as candidate ROCK inhibitors using the molecular hybridization of the clinically approved drug fasudil (**1**) and the IKK-β inhibitor LASSBio-1524 (**2**). Among the synthesized compounds, the *N*-methylated derivative **11** (LASSBio-2065) showed the best inhibitory profile for both ROCK isoforms, with IC_50_ values of 3.1 and 3.8 µM for ROCK1 and ROCK2, respectively. Moreover, these compounds were also active in the scratch assay performed in human breast cancer MDA-MB 231 cells and did not display toxicity in MTT and LDH assays. Molecular modelling studies provided insights into the possible binding modes of these *N*-sulphonylhydrazones, which present a new molecular architecture capable of being optimized and developed as therapeutically useful ROCK inhibitors.

## Introduction

Rho kinases, also called ROCKs (Rho-associated protein kinases), were the first Rho GTPases effector systems to be discovered. ROCKs are serine/threonine kinases belonging to the AGC family of protein kinases and were initially characterized by their roles in the formation of stress fibres induced by RhoA and focal adhesions by phosphorylating myosin light chain^1^.

These proteins coexist in two isoforms, ROCK1 (ROKβ or p160ROCK) and ROCK2 (ROKα), which share 65% homology at the primary sequence level and approximately 92% homology within the ATP binding sites^2^. These proteins are comprised of an *N*-terminal lobe containing a highly conserved Ser/Thr kinase domain, a coiled coil structure, a pleckstrin domain, and a cysteine-rich domain at the C-terminal lobe, which is connected by a hinge region^3^. These proteins typically form a self-inhibitory complex that is disrupted upon the binding of the activated Rho-GTP protein to the Rho binding domain (RBD), leading to kinase activation^3^.

Initially, ROCK1 was reported to be primarily distributed in the lungs, liver, spleen, kidneys and testes, and ROCK2 in neuronal tissues, skeletal muscles, heart, and placenta^4^. However, in the study published by Linda and Olson in 2014, the distributions of ROCK isoforms were similar, based on observations of EST markers, and little specificity in expression between organs or tissues was observed. ROCK1 is expressed at high levels in the thymus and blood, whereas little or no ROCK2 expression was observed^2^. In addition to the tissue distribution, these protein kinases have different intracellular distribution patterns: ROCK2 is predominantly cytosolic and ROCK1 is mainly associated with centrosomes and the plasma membrane^5^^,^^6^.

Activation of ROCKs by GTPases, mainly Rho, or by alternative pathways involving caspases or lipid mediators leads to the phosphorylation of several molecular targets. One of the main substrates of ROCK-mediated phosphorylation is MLC (myosin light chain), since the activation of these proteins was initially reported to be associated with the formation of stress fibres and changes in the cytoskeleton^4^^,^^7^. In this manner, ROCKs directly phosphorylate myosin light chain, promoting the actin-myosin interaction. In addition, ROCKs phosphorylate and inactivate MLCP (myosin light chain phosphatase), indirectly regulating the amount of phosphorylated myosin^4^^,^^8^.

The Rho/ROCK pathway is an important regulator of vascular smooth muscle cell contraction and is important in controlling migration, proliferation, differentiation, apoptosis, survival, and transcription^9^. Despite the growing interest of the major pharmaceutical and small biotechnology companies in the Rho/ROCK pathway and the reported pleiotropic effects of the ROCK inhibitors, relatively few inhibitors that have reached clinical trials or even the market^3^. The most frequent indications for the clinical use of ROCK inhibitors are in the field of cardiovascular diseases^10^, since systemic exposure to ROCK inhibitors promotes a rapid and pronounced decrease in blood pressure. A critical challenge is therefore to differentiate the desired pharmacological activity and adverse cardiovascular effects, since these adverse effects are not tolerated in most cases, as well as to obtain selectivity for ROCK isoforms^11^.

The first described ROCK inhibitor, i.e. the isoquinoline derivative fasudil (**1**), has been applied in the clinic as a treatment for subarachnoid haemorrhage (SAH) in Japan since 1995^12^^,^^13^. Fasudil exerts its effects by upregulating endothelial nitric oxide synthase (eNOS) expression and decreasing smooth muscle spasms and the migration of inflammatory cells^14^. Moreover, as shown in our recent study, the inhibition of ROCK pathway with fasudil induces the nuclear translocation of beta-catenin to reduce MDA MB-231 tumour cell migration^15^.

Previously, our research group proposed the use of the privileged *N*-acylhydrazone (NAH) framework^16^ as a starting point for structural modifications, leading to the discovery of novel Ser/Thr kinase inhibitors^17–19^. Accordingly, the prototype LASSBio-1524 (**2**) was discovered using structure-based drug design studies and showed a moderate IKK-β inhibition profile^17^.

In general, protein kinases that belong to the same classes have highly conserved domains, indicating that they possess high structural similarity in the catalytic site. Based on the previous study published by Avila and coworkers on the use of NAH compounds as IKK-β inhibitors^16^ and considering that ROCKs are also serine–threonine kinases, we propose here a series of novel molecular hybrids between the well-known ROCK inhibitor fasudil and LASSBio-1524, an IKK-β inhibitor. The proposed hybrids (**5a–h**) presenting novel molecular architectures are expected to participate in additional interactions with ROCK that may increase the molecular recognition profile in the ROCK active site.

**Figure 1.**
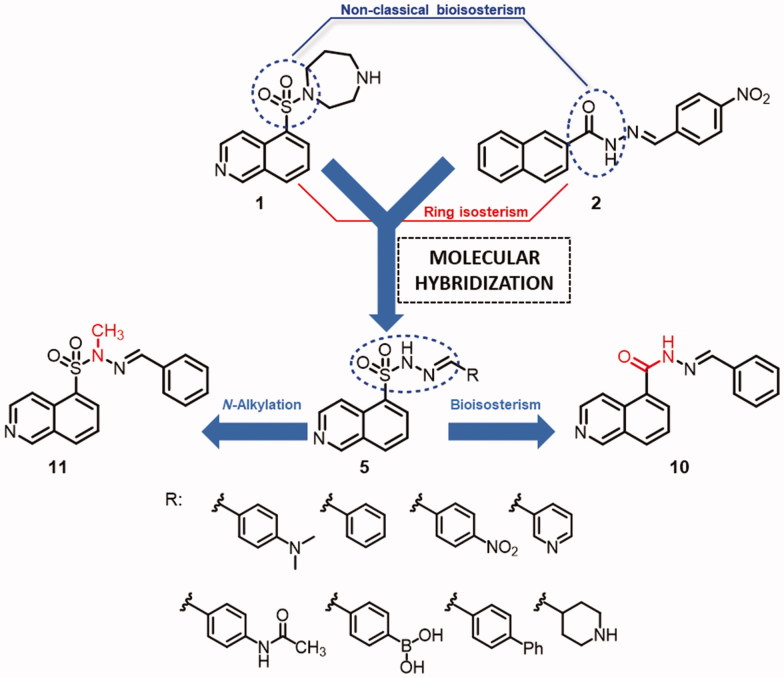
Design concept used to generate a novel class of *N*-sulphonylhydrazones as potential ROCK inhibitors.

During the design of the novel hybrid analogues (**5a–h**) presented in Figure 1, we exploited the isosteric relationship between the amide subunit of the *N*-acylhydrazone moiety present in LASSBio-1524 (**2**) and the sulphonamide unit present in fasudil (**1**). We have retained the isoquinoline ring substituted at the 5 position, because it is necessary for the molecular recognition of fasudil in the ROCK active site, and the *N*-sulphonylhydrazone fragment was incorporated in the hybrids (**5a–h**).

**Figure 2.**
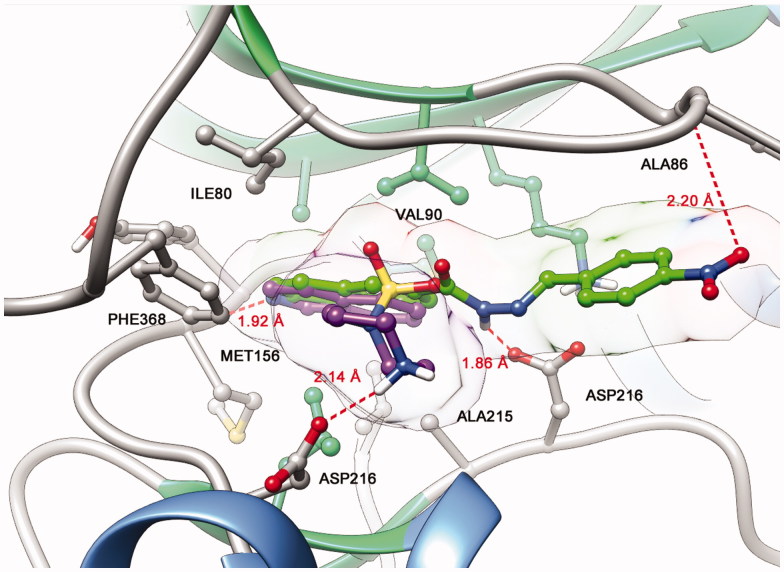
Representation of ROCK (PDB code: 2ESM) with the co-crystallized isoquinoline inhibitor fasudil (**1**) (magenta) and best interaction mode of LASSBio-1524 (**2**) (green) obtained through docking studies.

The proposed structural design was additionally supported by docking studies of LASSBio-1524 (**2**) and fasudil (**1**) at the active site of ROCK, which confirmed the importance of introducing the *N*-sulphonylhydrazone framework in the molecular architecture of target compounds (**5a–h**) (Figure 2).

The imine substituents were chosen according to the substitution pattern present in fasudil (**1**) and LASSBio-1524 (**2**) prototypes, exploring classical and non-classical isosteric relationships, as well as the simplification of the homopiperazine ring to evaluate its effect on molecular recognition in the active site of ROCK isoforms and subsequent inhibition. In addition, we also proposed the synthesis of an isoquinolinyl-*N*-acylhydrazone analogue to rationalize the effects of the bioisosteric exchange between sulphonamide and amide functional groups, as well as an *N*-methylated analogue to analyse the effect of *N*-alkylation on the kinase inhibitory activity.

## Experimental

### General information

The melting points of the intermediates were determined using a Quimis 340 apparatus. Hydrogen NMR spectra were determined in deuterated chloroform or dimethylsulphoxide containing approximately 1% tetramethylsilane (TMS) as an internal standard using a Bruker DPX-200 at 200 MHz, DRX-300 at 300 MHz, Varian 400-MR at 400 MHz and Varian 500-MR at 500 MHz. Carbon NMR spectra were determined using the same spectrometer at 50, 75, 100, and 125 MHz, respectively, and employing the same solvents. IR spectra (cm^−1^) were obtained using a Thermo Scientific Nicolet module Smart ITR. The progress of all reactions was monitored through thin-layer chromatography performed on 2.0 × 6.0 cm aluminium sheets precoated with silica gel 60 (HF-254, Merck) to a thickness of 0.25 mm. The developed chromatograms were viewed under ultraviolet light (254−366 nm) and treated with iodine vapour. The reagents and solvents were purchased from commercial suppliers and used as received. Analytical HPLC was performed for compound purity determinations using a Shimadzu LC-20AD with a Kromasil 100–5C18 column (4.6 × 250 mm) and a Shimadzu SPD-M20A detector. The solvent system used for the HPLC analyses was acetonitrile:water at 60:40 with or without of 0.5% trifluoracetic acid. The isocratic HPLC mode was used, and the flow rate was 1.0 ml/min. The purity of the compounds was higher than 95%. Ultraviolet spectroscopy was performed using a Femto spectrophotometer. The wavelength used in the solubility assay was determined as the maximum λ characteristic of each compound. The spectra were analysed using the Femto Scan software. High resolution mass spectrometry was performed through positive or negative ionization by Solarix XR 7Tesla, using ESI or APCI FTICR and the data were analysed using the mMass 5.5.0 software.

### Synthesis of isoquinoline-5-sulphonohydrazide (4)

In a 125-ml erlenmeyer flask, isoquinoline-5-sulphonyl chloride hydrochloride (**3**) (0.5 g, 1.89 mmol) was slowly added to a saturated sodium bicarbonate solution. The mixture kept its pH at a constant value (pH 5–6), was stirred for 30 min, then extracted with 2 × 30 ml of DCM. The organic layer was dried using magnesium sulphate and concentrated. The residue was re-dissolved in 2 ml of DCM and drop wise added to a solution of hydrazine hydrate 100% (5 equiv) in DCM, maintaining the temperature between –5 and –10 °C. The reaction mixture was stirred for 5h at room temperature (completion of the reaction was monitored by TLC), and after the completion, the solvent was removed under vacuum and the crude product was poured into ice flakes. The white precipitate was filtered as a white solid (365 mg, 87% yield); mp: 150 °C. ^1^H NMR (400 MHz, DMSO-d6) δ (ppm): 9.47 (s, 1H); 8.82 (s, 1H); 8.67 (d, *J* = 8.67 Hz, 1H); 8.47 (d, *J* = 8.48 Hz, 1H); 8.45 (d, *J* = 8.45 Hz, 1H); 8.37 (d, *J* = 8.37 Hz, 1H); 7.85 (t, *J* = 8.45 Hz, 1H); 4.26 (s, 2H). ^13^C NMR (100 MHz, DMSO-d6) δ (ppm): 153.1; 144.2; 134.4; 133.7; 132.7; 130.9; 128.8;126.3; 117.7. IR (ATR, cm^−1^): 3376; 3260; 3175; 1616; 1300; 1188. LRMS (ESI, m/z): calculated for [M + H]^+^ C_9_H_9_N_3_O_2_SH^+^, 224.05, found 223.85.

### General procedure for the synthesis of N-sulphonylhydrazones (5a–h)

A mixture of the required isoquinoline-5-sulphonohydrazide (**4**) (1 mmol) and the appropriate aldehyde (1 mmol) were dissolved in ethyl alcohol (15 ml) and vigorously stirred for 30–120 min at room temperature. Once the reaction was completed, (reaction was monitored by TLC), the solvent was vacuum removed, and the product precipitated by the drop wise addition of cold water. Following filtration, the precipitate was washed with H_2_O (10 ml) and *n*-hexane (10 ml) and dried in vacuum.

### (E)-N′-(4-(dimethylamino)benzylidene)isoquinoline-5-sulphonohydrazide (5a) – (LASSBio-2019)

Compound **5a** (LASSBio-2019) was obtained as an orange solid through condensation of isoquinoline-5-sulphonohydrazide (**4**) and 4-(dimethylamino)benzaldehyde (318.6 mg, 90% yield); mp: 165 °C. ^1^H NMR (400 MHz, DMSO-d6) δ (ppm): 11.53 (s, 1H); 9.44 (s, 1H); 8.74 (d, *J* = 6.1 Hz, 1H); 8.67 (d, *J* = 6.1 Hz, 1H); 8.47 (d, *J* = 8.4 Hz, 1H); 8.45 (d, *J* = 8.4 Hz, 1H); 7.87 (t, *J* = 7.8 Hz, 1H); 7.76 (s, 1H); 7.27 (d, *J* = 8.6 Hz, 2H); 6.61 (d, *J* = 8.6 Hz, 2H); 2.89 (s, 6H). ^13^C NMR (100 MHz, DMSO-d6) δ (ppm): 153.2; 151.4; 147.8; 144.3; 134.4; 133.9; 133.6; 130.7; 128.5; 128.0; 126.5; 120.7; 117.8; 111.6; 39.6. IR (ATR, cm^−1^): 2889; 2722; 1359; 1163. HRMS (ESI, m/z): calculated for [M + H]^+^ C_18_H_18_N_4_O_2_SH^+^, 355.1223, found 355.1225.

### (E)-N′-benzylideneisoquinoline-5-sulphonohydrazide (5b) – (LASSBio-2020)

Compound **5b** (LASSBio-2020) was obtained as a white solid through condensation of isoquinoline-5-sulphonohydrazide (**4**) and benzaldehyde (227.0 mg, 73% yield); mp: 172 °C. ^1^H NMR (400 MHz, DMSO-d6) δ (ppm): 12.06 (s, 1H); 9.48 (s, 1H); 8.75 (d, *J* = 8.7 Hz, 1H); 8.66 (d, *J* = 8.7 Hz, 1H); 8.51 (d, *J* = 8.5 Hz, 1H); 8.47 (d, *J* = 8.5 Hz, 1H); 7.89 (t, *J* = 7.8 Hz, 1H); 7.89 (s, 1H); 7.45–7.47 (m, 2H); 7.32–7.34 (m, 3H). ^13^C NMR (100 MHz, DMSO-d6) δ (ppm): 153.3; 151.4; 146.7; 144.3; 134.4; 133.9; 133.4; 130.1; 128.8; 126.7; 126.7; 117.8. IR (ATR, cm^−1^): 3536; 1332; 1157. HRMS (ESI, m/z): calculated for [M + H]^+^ C_16_H_13_N_3_O_2_SH^+^, 312.0801, found 312.0801.

### (E)-N′-(4-nitrobenzylidene)isoquinoline-5-sulphonohydrazide (5c) – (LASSBio-2021)

Compound **5c** (LASSBio-2021) was obtained as a yellow solid through condensation of isoquinoline-5-sulphonohydrazide (**4**) and 4-nitrobenzaldehyde as a yellow solid (267.0 mg, 75% yield); mp: 168 °C. ^1^H NMR (400 MHz, DMSO-d6) δ (ppm): 12.70 (s, 1H); 9.71 (s, 1H); 8.86 (s, 2H); 8.65 (d, *J* = 8.6 Hz, 1H); 8.62 (d, *J* = 8.6 Hz, 1H); 8.20 (d, *J* = 8.86 Hz, 2H); 8.06 (s, 1H); 8.02 (t, *J* = 7.8 Hz, 1H); 7.75 (d, *J* = 8.86 Hz, 2H). ^13^C NMR (100 MHz, DMSO-d6) δ (ppm): 150.5; 147.8. 144.7; 139.5; 138.0; 136.6; 136.0; 134.0; 132.4; 128.6; 128.3; 127.8; 124.0; 120.4. IR (ATR, cm^−1^): 2840; 1337; 1163. HRMS (ESI, m/z): calculated for [M + H]^+^ C_16_H_12_N_4_O_4_SH^+^, 357.0652, found 357.0653.

### (E)-N′-(pyridin-3-ylmethylene)isoquinoline-5-sulphonohydrazide (5d) – (LASSBio-2022)

Compound **5d** (LASSBio-2022) was obtained as a white solid through condensation of isoquinoline-5-sulphonohydrazide (**4**) and nicotinaldehyde (274.5 mg, 88% yield); mp: 168 °C. ^1^H NMR (400 MHz, DMSO-d6) δ (ppm): 9.81 (s, 1H); 8.95 (d, *J* = 6.5 Hz, 1H); 8.89 (d, *J* = 6.5 Hz, 1H); 8.76 (d, *J* = 7.8 Hz, 1H); 8.72 (t, *J* = 7.8 Hz, 1H); 8.70 (d, *J* = 8 Hz, 1H); 8.40 (d, *J* = 8 Hz, 1H); 8.13 (s, 1H); 8.90 (s, 1H); 8.07 (t, *J* = 7.8 Hz, 1H); 7.84 (dd, J1 = 8 Hz, J2 = 8 Hz, 1H). ^13^C NMR (100 MHz, DMSO-d6) δ (ppm): 152.7; 148.4; 146.3; 143.1; 142.9; 135.5. 134.9; 134.6; 133.0; 131.0; 130.3; 128.5; 127.1; 124.8; 118.2. IR (ATR, cm^−1^): 2637; 2722; 1376; 1168. HRMS (ESI, m/z): calculated for [M + H]^+^ C_15_H_12_N_4_O_2_SH^+^, 313.0754, found 313.0754.

### (E)-N-(4-((2-(isoquinolin-5-ylsulphonyl)hydrazono)methyl)phenyl)acetamide (5e) – (LASSBio-2023)

Compound **5e** (LASSBio-2023) was obtained as a yellow solid through condensation of isoquinoline-5-sulphonohydrazide (**4**) and 4-acetamidobenzaldehyde (312.8 mg, 85% yield); mp: 173 °C. ^1^H NMR (400 MHz, DMSO-d6) δ (ppm): 11.86 (s, 1H); 10.07 (s, 1H); 9.45 (s, 1H); 8.75 (d, *J* = 6.1 Hz, 1H); 8.63 (d, *J* = 6,1 Hz, 1H); 8.47 (t, *J* = 8.4 Hz, 2H); 7.88 (t, *J* = 7.8 Hz, 1H); 7.81 (s, 1H); 7.55 (d, *J* = 8.5 Hz, 2H); 7.38 (d, *J* = 8.5 Hz, 2H); 2.02 (s, 3H). ^13^C NMR (100 MHz, DMSO-d6) δ (ppm): 168.6; 153.4; 146.6; 144.6; 141.1; 134.3; 133.7; 133.7; 130.7; 128.5; 128.1; 127.5; 126.6; 124.1; 118.8; 117.7. IR (ATR, cm^−1^): 3898; 3216; 1663; 1321; 1158. HRMS (ESI, m/z): calculated for [M + H]^+^C_18_H_16_N_4_O_3_SH^+^, 369.1016, found 369.1015.

### (E)-(4-((2-(isoquinolin-5-ylsulphonyl)hydrazono)methyl)phenyl)boronic acid (5f) – (LASSBio-2024)

Compound **5f** (LASSBio-2024) was obtained as a white solid through condensation of isoquinoline-5-sulphonohydrazide (**4**) and 4-(dihydroxyboryl)benzaldehyde (273.0 mg, 77% yield); mp: >250 °C. ^1^H NMR (400 MHz, DMSO-d6) δ (ppm): 12.06 (s, 1H); 9.48 (s, 1H); 8.77 (d, *J* = 5.7 Hz, 1H); 8.67 (d, *J* = 5.7 Hz, 1H); 8.51 (d, *J* = 8.0 Hz, 1H); 8.47 (d, *J* = 8.0 Hz, 1H); 7.90 (t, *J* = 8.0 Hz, 1H); 7.90 (s, 1H); 7.73 (d, *J* = 7.4 Hz, 2H); 7.42 (d, *J* = 7.4 Hz, 2H). ^13^C NMR (100 MHz, DMSO-d6) δ (ppm): 153.2; 146.8; 144.0; 134.8; 134.5; 134.4; 134.0; 133.7; 133.7; 130.8; 128.5; 126.8; 125.7; 117.9. IR (ATR, cm^−1^): 1325; 1161. HRMS (ESI, m/z): calculated for [M + H]^+^ C_16_H_14B_N_3_O_4_SH^+^, 356.0871, found 356.0871.

### (E)-N′-([1,1'-biphenyl]-4-ylmethylene)isoquinoline-5-sulphonohydrazide (5g) – (LASSBio-2025)

Compound **5g** (LASSBio-2025) was obtained as a yellow solid through condensation of isoquinoline-5-sulphonohydrazide (**4**) and [1,1'-biphenyl]-4-carbaldehyde (313.4 mg, 81% yield); mp: 166 °C. ^1^H NMR (400 MHz, DMSO-d6) δ (ppm): 12.52 (s, 1H); 9.91 (s, 1H); 9.10 (d, *J* = 6.5 Hz, 1H); 8.92 (d, *J* = 6.5 Hz, 1H); 8.76 (t, *J* = 7.8 Hz, 2H); 8.13 (t, *J* = 7.8 Hz, 1H); 8.05 (s, 1H); 7.62–7.67 (m, 4H); 7.57 (d, *J* = 8 Hz, 2H); 7.44 (t, *J* = 7.5 Hz, 2H); 7.35 (t, *J* = 7.4 Hz, 1H). ^13^C NMR (100 MHz, DMSO-d6) δ (ppm): 149.9; 147.0; 141.7; 139.1; 137.2; 136.5; 136.1; 134.4; 132.9; 132.4; 129.0; 128.9; 128.2; 127.9; 127.5; 127.0; 126.6; 121.2. IR (ATR, cm^−1^): 2736; 1336; 1165. HRMS (ESI, m/z): calculated for [M + H]^+^ C_22_H_17_N_3_O_2_SH^+^, 388.1114, found 388.1114.

### (E)-tert-butyl 4-((2-(isoquinolin-5-ylsulphonyl)hydrazono)methyl)piperidine-1-carboxylate (6)

Compound **6** was obtained as yellow oil through condensation of isoquinoline-5-sulphonohydrazide (**4**) and tert-butyl 4-formylpiperidine-1-carboxylate (347.0 mg, 83% yield). ^1^H NMR (400 MHz, DMSO-d6) δ (ppm): 11.49 (s, 1H); 9.47 (s, 1H); 8.68 (d, *J* = 6 Hz, 1H); 8.52 (d, *J* = 6 Hz, 1H); 8.47 (d, *J* = 8.0 Hz, 1H); 8.39 (d, *J* = 8.0 Hz, 1H); 7.86 (t, *J* = 8.0 Hz, 1H); 7.16 (d, *J* = 4 Hz, 1H); 3.63–3.66 (m, 2H); 2.59–2.72 (m, 2H); 2.18–2.24 (m, 1H); 1.38 (s, 9H); 1.36–1.48 (m, 2H); 0.99–1.07 (m, 2H). LRMS (ESI, m/z): calculated for [M + H]^+^ C_20_H_26_N_4_O_4_SH^+^, 419.17, found 419.09.

### (E)-N′-(piperidin-4-ylmethylene)isoquinoline-5-sulphonohydrazide hydrochloride (5h) – (LASSBio-2055)

In a 50 ml round bottom flask, 300 mg of **6** (0.7 mmol) was dissolved in 15 ml of dry ethanol. Then, 1.570 mg of acetyl chloride (14 mmol) was added drop wise to a stirred solution of the *N*-(tert-butoxycarbonyl)-protected sulphonylhydrazone (**6**) at room temperature. The mixture was stirred overnight and evaporated in vacuum to give the title compound as a pale yellow solid, which was recrystallized in methanol (156 mg, 63% yield); mp: 186 °C.^1^ H NMR (400 MHz, DMSO-d6) δ (ppm): 12.07 (s, 1H); 9.92 (s, 1H); 8.95 (d, *J* = 6 Hz, 1H); 8.88 (d, *J* = 6 Hz, 1H); 8.75 (d, *J* = 8 Hz, 1H); 8.65 (d, *J* = 8 Hz, 1H); 8.10 (t, *J* = 8 Hz, 1H); 7.30 (d, *J* = 4 Hz, 1H); 3.05–3.07 (m, 2H); 2.74–2.81 (m, 2H); 2.34–2.40 (m, 1H); 1.69–1.72 (m, 2H); 1.39–1.47 (m, 2H). ^13^C NMR (50 MHz, DMSO-d6) δ (ppm): 153.2; 152.6; 149.9; 136.8; 136.0; 134.5; 132.9; 128.9; 128.2; 121.0; 42.2; 35.5; 25.0. IR (ATR, cm^−1^): 2949; 2695; 1327; 1179. HRMS (ESI, m/z): calculated for [M + H]^+^ C_15_H_18_N_4_O_2_SH^+^, 319.1223, found 319.1223.

### (E)-N′-benzylidene-N-methylisoquinoline-5-sulphonohydrazide (11) – (LASSBio-2065)

In a 25-ml reaction flask, 150 mg of **5b** (LASSBio-2020) (0.48 mmol), 10 ml of acetone, 199 mg of potassium carbonate (K_2_CO_3_) (1.44 mmol) and 0.1 ml of methyl iodide (CH_3_I) (1.44 mmol) were solubilized. The flask was coupled to a condenser and the reaction was kept under reflux and constant stirring overnight. The acetone volume was reduced in a rotary evaporator, and the product purified by flash column chromatography to give a yellow solid (109.3 mg, 70% yield); mp: 108 °C. ^1^H NMR (400 MHz, DMSO-d6) δ (ppm): 9.42 (s, 1H); 8.71–8.75 (m, 2H); 8.47–8.51 (m, 2H); 7.90 (m, 1H); 7.79 (s, 1H); 7.55–7.57 (m, 2H); 7.33–7.41 (m, 3H); 3.30 (s, 3H). ^13^C NMR (100 MHz, DMSO-d6) δ (ppm): 153.3; 144.6; 143.7; 134.9; 134.7; 133.9; 131.0; 131.0; 130.0; 128.8; 128.6; 127.0; 126.6; 118.0; 32.9. IR (ATR, cm^−1^): 1364; 1162. HRMS (ESI, m/z): calculated for [M + H]^+^ C_17_H_15_N_3_O_2_SH^+^, 326.0958, found 326.0958.

### Methyl isoquinoline-5-carboxylate (8)

In a 125 ml flask, methanolic solutions (each 10 ml) of iodine (2.098 g, 8.26 mmol) and KOH (926 mg, 16.5 mmol) at 0 °C were successively added to a solution of isoquinoline-5-carboxaldehyde (**7**) (1000 mg, 6.36 mmol) in 50 ml of absolute methanol cooled to 0 °C. After stirring for 10 h at 0 °C, 50 ml of saturated NaHSO_3_ solution was added to the reaction, resulting in the disappearance of the brown colour. The methanol was then evaporated under reduced pressure. The remaining content was stirred for 30 min at 0 °C, and the title compound was obtained through filtration as a beige solid (1070 mg, 90% yield); mp: 66 °C. ^1^H NMR (400 MHz, CDCl_3_): 9.33 (s, 1H); 8.82 (d, *J* = 6 Hz, 1H); 8.61 (d, *J* = 6 Hz, 1H); 8.46 (d, *J* = 7 Hz, 1H); 8.19 (d, *J* = 7 Hz, 1H); 7.65 (t, *J* = 7 Hz, 1H); 3.99 (s, 3H). ^13^C NMR (100 MHz, CDCl_3_): 166.6; 152.4; 143.6; 135.2; 134.5; 133.5; 128.8; 126.5; 125.8; 119.1; 52.5.

### Isoquinoline-5-carbohydrazide (9)

In a 50-ml flask was added 500 mg of methyl isoquinoline-5-carboxylate (**8**) (2.67 mmol), 15 ml ethanol and 670 mg hydrazine hydrate (100%) (13.35 mmol). The reaction mixture was kept stirring under reflux overnight. After the completion of the reaction, ethanol was concentrated under reduced pressure, leading to the formation of a pale yellow solid (434.3 mg, 87% yield); mp: 172 °C. ^1^H NMR (400 MHz, DMSO-d6) δ (ppm): 9.80 (s, 1H); 9.37 (s, 1H); 8.55 (d, *J* = 6 Hz, 1H); 8.23 (d, *J* = 7 Hz, 1H); 8.11 (d, *J* = 6 Hz, 1H); 7.86 (d, *J* = 7 Hz, 1H); 7.71 (t, *J* = 7 Hz, 1H); 4.62 (s, 2H). IR (ATR, cm^−1^): 3216; 3002; 1643. LRMS (ESI, m/z): calculated for [M + H]^+^ C_10_H_9_N_3_OH^+^, 188.08, found 188.02.

### (E)-N′-benzylidene isoquinoline-5-carbohydrazide (10)

Compound **10** was obtained as a white solid through condensation of isoquinoline-5-carbohydrazide (**9**) and benzaldehyde (223 mg, 81% yield), following the same general procedure used for the synthesis of *N*-sulphonylhydrazones (**5**). ^1^H NMR (400 MHz, DMSO-d6) δ (ppm): 12.08 (s, 1H); 9.41 (s, 1H); 8.59 (d, *J* = 6 Hz, 1H); 8.38 (s, 1H); 8.32 (d, *J* = 7 Hz, 1H); 8.14 (d, *J* = 6 Hz, 1H); 8.08 (d, *J* = 7 Hz, 1H); 7.74–7.82 (m, 3H); 7.45–7.52 (m, 2H); 7.20–7.30 (m, 1H). ^13^C NMR (100 MHz, DMSO-d6) δ (ppm): 163.5; 152.6; 147.9; 143.7; 134.1; 133.6; 132.6; 130.5; 130.3; 130.1; 128.6; 128.4; 127.4; 126.5; 117.8. IR (ATR, cm^−1^): 1635. HRMS (APCI, m/z): calculated for [M + H]^+^ C_17_H_13_N_3_OH^+^, 276.1131, found 276.1129.

### Water solubility assay

The solubility assay was performed considering the absorptivity of compounds under ultraviolet spectroscopy as described by Schneider *et al*.^20^ Assay wavelength was determined through λ_max_ characteristic of each compound. Saturated aqueous solutions were prepared (0.8–1.0 mg/mL) and kept under stirring for 4 h at 37 °C. The supernatant was filtered in 0.45 mm filters and transferred to a quartz cuvette (10 mm) for spectra acquisition. Solubility was determined by linear regression used as Excel graph plots, solutions were prepared by dilutions of the original solution in methanol. Data were collected in triplicate and the mean values were used to create the graph plots. The correlation coefficient (*R*^2^) values were between 0.9982 and 1.

### Chemical stability study

Two microliters (0.01 mmol) of a concentrated solution of the compound under examination (40 mM stock solution solubilized in DMSO) and 248 μL of acid (0.2 M potassium chloride and 0.2 M HCl; pH 2.0) or neutral (phosphate dibasic, pH 7.4) buffer were added to a 2 ml Eppendorf microtube. After vortexing, the mixture was placed in a water bath at 37 °C under vigorous stirring for 0, 30, 60, 120, and 240 min. After each reaction, 248 μL of basic buffer (phosphate buffer, pH 8.4) was added to neutralize the pH of the medium in the experiments using acidic buffer. The compound was extracted using 1 ml of acetonitrile followed by vigorous vortexing and freezing of the aqueous phase (−10 °C). The organic phase was separated, filtered, and analysed using an HPLC-PDA (acetonitrile/water 60:4, 0.05% of TFA).

### Molecular modelling

The ligands used for molecular docking studies were drawn and their 3 D geometry optimized using the semi-empirical method PM6 implemented in GAUSSIAN09W. We searched for co-crystals containing ligands with some structural similarity to the tested compounds and the model used was ROCK1 structure complexed with fasudil (PDB: 2ESM, resolution: 2.20 Å).The four fitness functions available in the GOLD 5.4.1 program, namely (ASP, ChemPLP, ChemScore and GoldScore) were evaluated for the redocking of the co-crystallized ligand to identify the most adequate fitness function for the docking studies. Crystallographic water molecules were removed during the docking runs, and the binding site was determined within 7 Å within the active site. The ChemPLP fitness function was the best reproducing the binding pose as the crystal structure (RMSD 0.2798 Å) and then used to perform the docking studies. Molecular graphics and analysis were made with the UCSF Chimera package^21^ and 2 D representations with LigPlot^+^.^22^

### X-ray diffraction

In order to get a suitable powder for the X-ray diffraction data collection **5f** (LASSBio-2024) sample was gently hand-grinded in an agate mortar and then packed between two 0.014-mm thick cellulose/acetate foils in a sample holder that was held spinning during data collection. The data was recorded at room temperature on a STADI-P powder diffractometer, from Stoe^®^ (Darmstadt, Germany) in transmission geometry by using Cu Kα_1_ radiation (λ = 1.54056 Å) and selected by a curved monochromator Ge (111), with a tube voltage of 40 kV and a current of 40 mA. The intensities were collected by a silicon microstrip detector, Mythen 1 K (Dectris^®^, Baden, Switzerland), in the range from 3° to 91.185°, with steps of 0.015° and a counting time of 200 s at each 1.05°.

### ROCK inhibition

The inhibition of ROCK isoforms was made in EUROFINS-CEREP France (www.cerep.fr) under the following study numbers: 100033685 and 100040481, using the protocol previously published by Doe *et al*.^23^ and Turner *et al*.^24^, respectively, for ROCK1 and ROCK2 human recombinant enzymes. The analysis was performed using software developed at Cerep (Hill software) and GraphPad Prism^®^ 5.0 (GraphPad Software, Inc., San Diego, CA) and validated through comparison with results generated using the SigmaPlot 4.0 software.

### Cell culture

The human mammary gland/breast epithelial cell line MDA-MB 231 was obtained from the American Type Culture Collection (ATCC^®^ HTB-26^™^) and this use was approved by the Ethics Committee for Animal Care and Use in Scientific Research from the Federal University of Rio de Janeiro. Cells were routinely grown in RPMI medium containing 10% foetal bovine serum (FBS), 1% L-glutamine and 1% penicillin-streptomycin, in a humidified 5% CO_2_ atmosphere at 37 °C. Cells were cultured up to 70–100% confluence and then some cultures were treated with the compounds at different concentrations for 24 or 48 h. All cell culture reagents were purchased from Invitrogen (São Paulo, Brazil).

### Cell viability assay

Cell viability was determined using 3–(4,5-dimethyl-2-thiazyl)-2,5-diphenyl-2H-tetrazolium bromide (MTT) reagent (Sigma-Aldrich). Briefly, cells were plated at an initial density of 2.5 × 104 cells per well in 96-well plates and incubated for 24 h at 37 °C and 5% CO_2_. After 24 h cultures were treated with **5b** (LASSBio-2020) and **11** (LASSBio-2065) at a final concentration of 0.1, 1, 10, 20, 30 μM and further incubated for 24 or 48 h. After treatment, the supernatant of each well was removed and cells were washed twice with medium. Then, 10 μl of MTT solution (5 mg/ml in RPMI) and 100 μl of medium were added to each well and incubated for 4 h at 37 °C, 5% CO_2_. The resultant formazan crystals were dissolved in dimethylsulphoxide (100 μl) and absorbance intensities were measured in a microplate reader (FlexStation Reader, Molecular Devices) at 570 nm. All experiments were performed in triplicate, and cell viability was expressed as a percentage relative to the untreated control cells.

### Plasma membrane integrity assay (LDH)

To measure plasma membrane integrity, we assayed serum lactate dehydrogenase (LDH) levels and calculated percentages of LDH release to the medium. LDH activity was measured spectrophotometrically using a commercial kit (Doles, Goiás, Brazil). After **5b** (LASSBio-2020) and **11** (LASSBio-2065) treatment, 50 μl of the supernatant were transferred to an enzymatic assay plate and 50 μL of LDH substrate plus 5 μL ferric albumen were added and incubated for 3 min at 37 °C, protected from light. Then, 10 μl of NAD were added and absorbance intensities were measured in a microplate reader (FlexStation^®^ 3 Reader, Molecular Devices) at 490 nm. The percentage of LDH release was calculated by ([LDH] sample ×100)/total [LDH]. [LDH] sample was the LDH level of the sample (released in medium) and total [LDH] was the LDH content in the wells after addition of lysis solution (0.9% Triton X-100).

### Cell-based scratch assay

Cells were cultured in 24-well culture plates for 24 h up to 90–100% confluence. Scratched wound lines were created with the help of a 200 μl micropipette tip. Wells were washed with RPMI for removal of non-adherent cells. Cells were incubated for 24 h with **5b** (LASSBio-2020) and **11** (LASSBio-2065) at a final concentration of 10 or 30 μM. All cell-based scratch assays were performed in the presence of the anti-mitotic reagent cytosine arabinoside (Arac; Sigma-Aldrich) at a final concentration of 10^−5 ^ M to inhibit cell proliferation. After **5b** (LASSBio-2020) and **11** (LASSBio-2065) treatment, the wound areas were observed with an Axiovert 100 microscope (Carl Zeiss, Germany). Images were acquired with an Olympus DP71 digital camera (Olympus, Japan) and the wound area was quantified using Fiji software (based on ImageJ, http://imageJ.nih.gov/ij/) from three different experiments.

## Results and discussion

### Chemistry

**Scheme 1.**
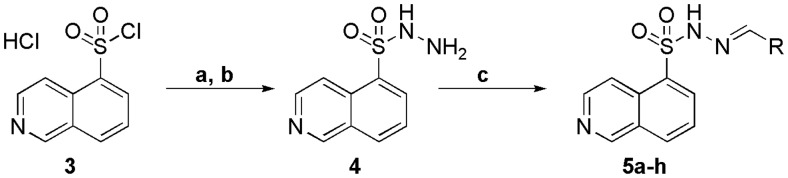
Synthetic route exploited to prepare the N-sulphonylhydrazones **(5a–h)**. a) NaHCO_3_ aqueous, dichloromethane; b) N_2_H_4_.H_2_O, dichloromethane, 0 °C, 4 h, 80%; c) EtOH, HCI (cat), r.t., 24 h, 70–90%.

The synthesis of the *N*-sulphonylhydrazone derivatives **5a–h** is outlined in Scheme 1. The starting material was the commercially available reagent isoquinoline-5-sulphonyl chloride hydrochloride (**3**), which was neutralized and condensed with hydrazine hydrate at 0 °C, furnishing the respective sulphonylhydrazide intermediate (**4**) at an 80% yield. Next, we performed the condensation of compound **4** with aromatic and non-aromatic aldehydes to obtain the corresponding *N*-sulphonylhydrazone derivatives **5a–h.**

**Scheme 2.**
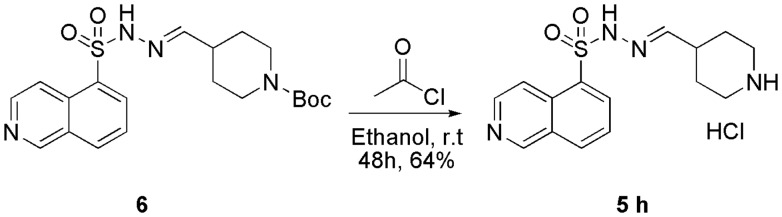
Synthesis of compound **5h** (LASSBio-2055) following the removal of N-Boc from derivative 6.

An additional step was performed to obtain the piperidinyl derivative **5h**, which consisted of the removal of the *N*-Boc group from *N*-sulphonylhydrazone derivative **6** (Scheme 2). For certain *N*-sulphonylhydrazones, the literature has reported the instability of the imine bond under acidic conditions, leading to hydrolysis.^25^ For this reason, a mild methodology was chosen to remove the protective group from compound **6**, avoiding the possible hydrolysis of the imine double bond, which could simultaneously result in the formation of the corresponding hydrochloride *in situ*. Therefore, we applied the protocol described by Nudelman and coworkers^26^ using acetyl chloride in ethanol to obtain the *N*-sulphonylhydrazone derivative **5h** at a 64% yield.

**Figure 3.**
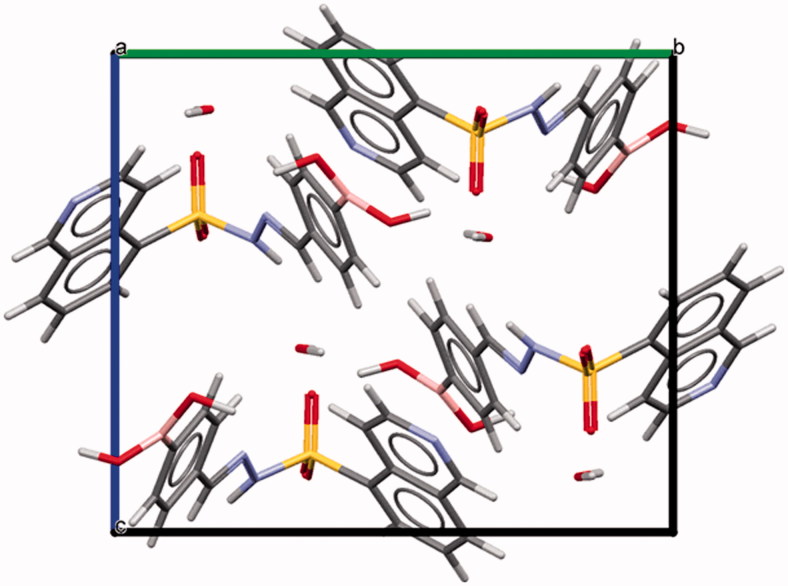
Unit cell representation of compound **5f** (LASSBio-2024) along the a-axis.

**Figure 4.**
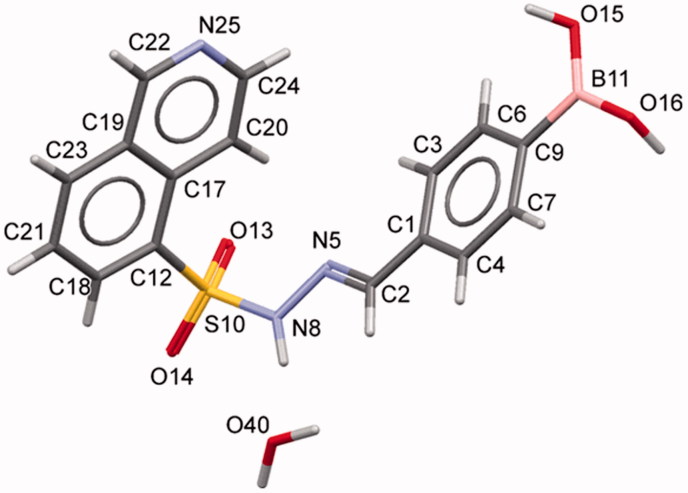
Crystal structure of compound **5f** (LASSBio-2024) showing the labels for all non-hydrogen atoms.

The condensation between hydrazides and aldehydes preferentially produces hydrazones with a relative (*E*) configuration.^27^ However, we performed X-ray diffraction studies to further determine which diastereomer was obtained. Due to the difficulty in obtaining all individual compounds in crystalline form, compound **5f** (LASSBio-2024) was chosen as representative of the series of *N*-sulphonylhydrazone derivatives. X-ray powder diffraction data were used to index the diffraction pattern and to determine the crystal structure of compound **5f** (LASSBio-2024). Similar detailed procedures have been reported elsewhere^28–31^. Briefly, compound **5f** (LASSBio-2024) crystallized as a monohydrate (C_16_H_14_BN_3_O_4_S⋅H_2_O) in a monoclinic crystal system—space group *P*2_1_/*c* (nr. 14). The unit cell comprises four formula units (*Z* = 4, shown in Figure 3) and one molecule in the asymmetric unit (*Z*’ = 1). We assigned the relative (*E*) configuration to the imine double bond (C = N)^32^. Within the unit cell, the molecules assumed a folded shape (Figure 4). Using PLATON^33^ and Mercury^34^^,^^35^ software, we confirmed the geometry of the molecule and the correct space group, unit cell parameters, bond distances, angles and torsions. CCDC ID: 1816869 contains the supplementary crystallographic data for this paper.

Considering that all *N*-sulphonylhydrazones were obtained using the same synthetic methodology, these experimental data were extrapolated to the other compounds of the series. Moreover, the similarity of the chemical shift of the imine functional group in the ^1^H NMR spectra using DMSO-d6 as solvent, as well as the absence of additional signals in the NMR spectra, corroborate the hypothesis that the compounds have been selectively obtained as (*E*) diastereoisomers. The chemical yields of the condensation step and HPLC purities are described in Table 1.

**Table 1.** *N*-Sulphonylhydrazones **5a**–**h** and their corresponding chemical yields and purities.

Compound	Formula	Molecular weight	Yields^a^ (%)	Purity^c^ (%)
**5a** (LASSBio-2019)	C_18_H_18_N_4_O_2_S	354.43	92	98
**5b** (LASSBio-2020)	C_16_H_13_N_3_O_2_S	311.36	73	97
**5c** (LASSBio-2021)	C_16_H_12_N_4_O_4_S	356.36	75	97
**5d** (LASSBio-2022)	C_15_H_12_N_4_O_2_S	312.35	88	96
**5e** (LASSBio-2023)	C_18_H_16_N_4_O_3_S	368.41	85	95
**5f** (LASSBio-2024)	C_16_H_14_BN_3_O_4_S	355.18	77	99
**5g** (LASSBio-2025)	C_22_H_17_N_3_O_2_S	387.45	81	99
**5h** (LASSBio-2055)	C_15_H_19_ClN_4_O_2_S	354.85	64^b^	99

^a^Yields of the condensation step.

^b^Cumulative yield of the condensation and deprotection steps.

^c^Determined by using reversed-phase HPLC analysis.

#### Biological evaluation

##### ROCK inhibition assay

The *N*-sulphonylhydrazone derivatives **5a–h** were evaluated for their ability to inhibit both ROCK1 and ROCK2 isoforms by measuring the phosphorylation of the Ulight-RRRSLLE substrate using human recombinant enzymes expressed in Sf9^23^ and Sf21^24^ cells, respectively. Prior to this assay, we evaluated the solubility of these compounds in water (buffer pH 7.4) to ensure that the inhibition percentages were not influenced by the precipitation of the compounds under test conditions. The enzymatic assay was initially performed at a screening concentration of 3 μM, which is capable of guaranteeing the solubility of most of the compounds, and the obtained results are shown in Table 2.

**Table 2.** ROCK inhibition profiles and aqueous solubility of *N*-sulphonylhydrazones **5a–h** and the standard compound fasudil (**1**).

Compound	% inhibition at 3 *μ*M		Aqueous solubility (µM)^b^
	ROCK1^a^	ROCK2^a^	
Fasudil	73.8	68.8	ND
**5a** (LASSBio-2019)	4.1	0.6	5.4
**5b** (LASSBio-2020)	29	24.2	54
**5c** (LASSBio-2021)	1.1	8.1	26
**5d** (LASSBio-2022)	4.5	7.6	>64
**5e** (LASSBio-2023)	4.6	−9.1	4.3
**5f** (LASSBio-2024)	2.7	7.3	58
**5g** (LASSBio-2025)	−1.1	2.4	<0.5
**5h** (LASSBio-2055)	6.9	3.9	>84.5

^a^Values are presented as averages of two experiments. Data are shown as % inhibition of ROCK.

^b^Determined by using the spectrophotometric method developed by Schneider and coworkers^20^. ND = Not determined.

Among the *N*-sulphonylhydrazone derivatives that were initially screened in the inhibition assays, only unsubstituted derivative **5b** (LASSBio-2020) showed a significant inhibitory profile at the screening concentration. Therefore, we decided to introduce additional modifications into this derivative to better understand the structure-activity relationships and to enhance the inhibitory profile towards ROCK isoforms.

**Scheme 3.**
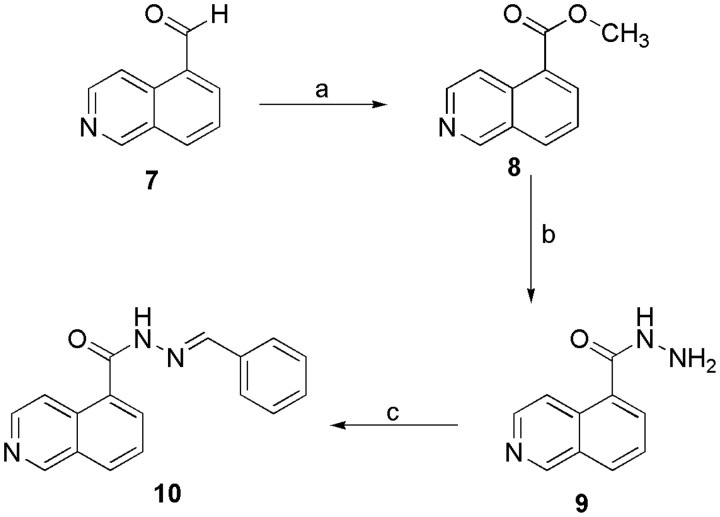
Synthetic route exploited to prepare the N-acylhydrazone derivative **10** (LASSBio-2064). a) KOH, I_2_, MeOH, 0 °C, 4h, 80%; b) N_2_H_4_.H_2_O, EtOH, reflux, overnight, 80%; c) EtOH, benzaldehyde, HCl (cat), overnight, 75%.

Initially, we investigated the bioisosteric replacement of the sulphonylhydrazone group in compound **5b** to an *N*-acylhydrazone group and proposed the synthesis of the *N*-acylhydrazone derivative **10** (LASSBio-2064). Employing the oxidative procedure reported by Yamada^36^, we converted the commercially available isoquinoline-5-carboxaldehyde (**7**) to the corresponding methyl ester (**8**) after treatment with 2.6 eq. of KOH and 1.3 eq. of iodine in methanol at 0 °C and obtained an 89% yield. Next, the key *N*-acylhydrazide intermediate (**9**) was obtained at a 70% yield by treating an ethanolic solution of the ester (**8**) with hydrazine hydrate under reflux^37^ (Scheme 3). The desired benzylidene-NAH derivative **10** (LASSBio-2064) was obtained at a 75% yield after condensing the hydrazide **9** with benzaldehyde in ethanol using hydrochloric acid as catalyst.

**Figure 5.**
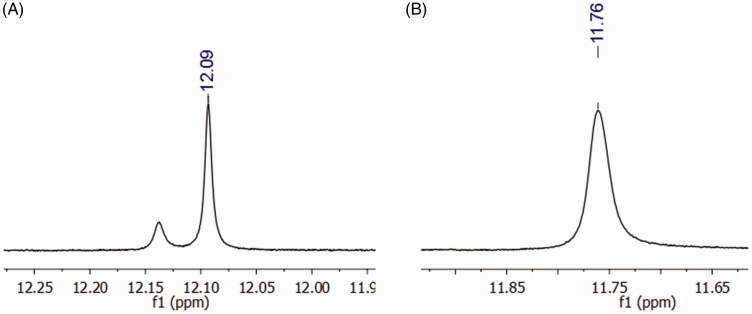
^1^H NMR shift of the amide proton of compound **10** (LASSBio-2064). (A) Experiment performed at 25 °C, where the duplication of the amide hydrogen was observed. (B) Experiment performed at 90 °C, where the coalescence of the signal was observed, indicating a conformational effect.

Although the derivative **10** (LASSBio-2064) presented adequate purity, as indicated by HPLC, duplicate signals in the ^1^H NMR spectrum at 12.09 ppm appeared. These additional signals might be due to a mixture of diastereoisomers or conformers. The hypothesis of diastereoisomers was excluded because only one singlet for the imine hydrogen was observed at 8.38 ppm. In addition, if interconversion between diastereoisomers occurred, the energy barrier for the interconversion of NAH (*E*) to (*Z*) is approximately 60 kcal/mol, which would be unfavourable in this case.^31^ An ^1^H NMR experiment with temperature variation was performed to completely discard the hypothesis of diastereomers. At 90 °C, the coalescence of the adjacent signal related to the amide nitrogen of the *N*-acylhydrazone group was observed (Figure 5); thus, we confirmed the presence of only one diastereoisomer and attributed this effect to the mixture of conformers at the amide bond. This phenomenon had already been observed for other NAH derivatives synthesized by the research group^27^.

**Scheme 4.**
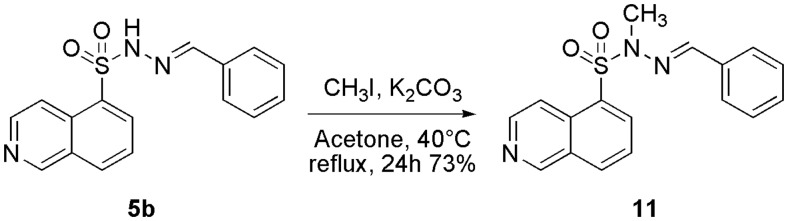
Synthesis of the N-methyl-N-sulphonylhydrazone derivative **11** (LASSBio-2065).

As suggested above, we also aimed to investigate the impact of the *N*-alkylation of the *N*-sulphonylhydrazone moiety on the biological activity, as previous results from our research group clearly indicated that this structural modification in *N*-acylhydrazones contributes to important changes in the conformational behaviour of these derivatives and produces subsequent important modifications in their bioactivity profiles^38^. Compound **5b** (LASSBio-2020) was employed as precursor of the *N*-alkylated derivative **11** (LASSBio-2065) and treated with potassium carbonate in acetone followed by the addition of methyl iodide at room temperature^37^. Under these conditions, we were able to obtain the *N*-methylated derivative **11** (LASSBio-2065) at a 73% yield (Scheme 4).

Next, we determined the IC_50_ values for compounds **5b** (LASSBio-2020), **10** (LASSBio-2064) and **11** (LASSBio-2065) against both ROCK isoforms in the presence of different concentrations ranging from 1 to 50 µM, which are within the limit of aqueous solubility previously determined for each compound. The percent enzyme activity was measured for each concentration to construct the inhibition curves (see the Supplementary Material) and thus determine the IC_50_ values shown in Table 3.

**Table 3.** Determination of IC_50_ values for *N*-sulphonylhydrazones **5b** (LASSBio-2020), **11** (LASSBio-2065) and the *N*-acylhydrazone **10** (LASSBio-2064) against human recombinant ROCK1 and ROCK2 enzymes.

Compound	IC_50_ (µM)	
	ROCK1^a^	ROCK2^a^
Fasudil (1)^b^	1.2	0.82
**5b** (LASSBio-2020)	13	7.1
**10** (LASSBio-2064)	—	—
**11** (LASSBio-2065)	3.1	3.8

^a^The IC_50_ values displayed above are the means of two experiments ± standard deviations presented in μM. Compounds were examined using a five-point enzyme assay.

^b^The IC_50_ values were previously determined^39^.

**Figure 6.**
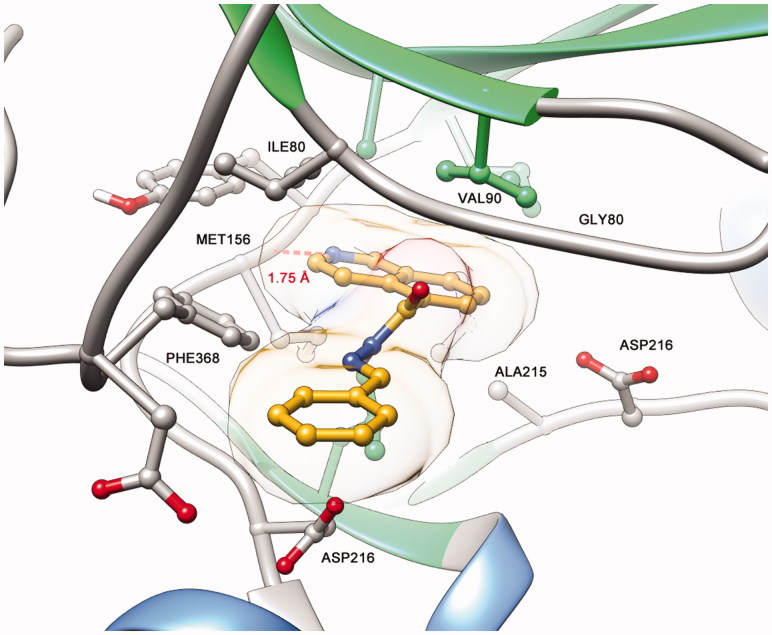
Predicted binding mode of compound **11** (LASSBio-2064) in complex with ROCK. Docking studies were performed using the program GOLD 5.4.1.

**Figure 7.**
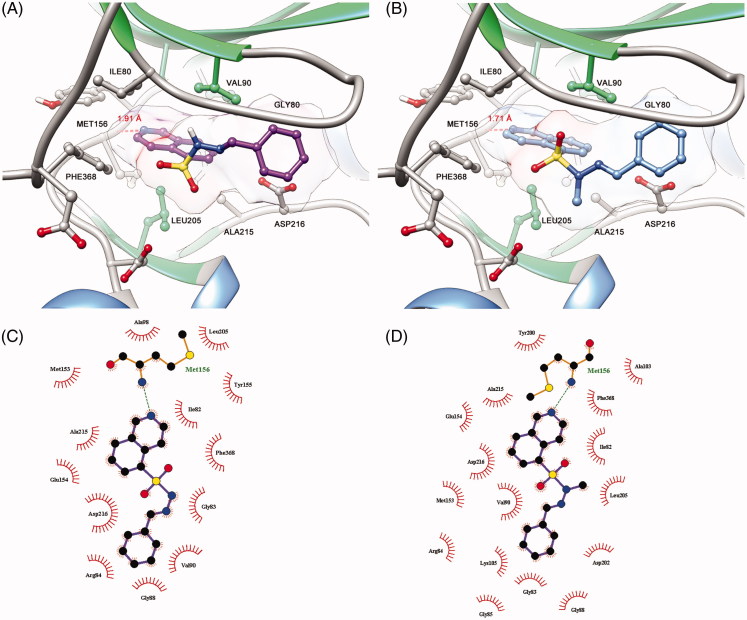
(A) Predicted binding mode of compound **5b** (LASSBio-2020) in complex with ROCK. (B) Predicted binding mode of compound **11** (LASSBio-2065) in complex with ROCK. (C) Ligplot 2D representation of compound **5b** (LASSBio-2020). (D) Ligplot 2D representation of compound **11** (LASBio-2065).

The derivative **10** (LASSBio-2064) was completely inactive in the ROCK inhibition assays. Apparently, the conformation adopted at the active site of target enzymes promoted by the bioisosteric exchange of the sulphone group for the carbonyl group disfavoured molecular recognition at the catalytic sites of ROCK. Additionally, as shown in Figure 6, compound **10** (LASSBio-2064) had a different mode of interaction from the *N*-sulphonylhydrazone congener series (Figure 7). Apparently, the conformation adopted in the active site promoted by the bioisosteric exchange of the sulphone group for the carbonyl group disfavoured molecular recognition in the catalytic sites of ROCK isoforms. The rotation of the carbonyl group caused the substituent bound to the imine functional group to interact with another hydrophobic cavity of the active site that differed from the hydrophobic cavity used by the *N*-sulphonylhydrazone analogues. This mode of interaction seems to be unfavourable and was corroborated by the study published by Chen *et al*.^14^, who reported modifications in the structure of fasudil (**1**) and revealed the pharmacophoric nature of the sulphonyl group in relation to the respective carbonyl isostere.

In addition, we determined the IC_50_ values for compounds **5b** (LASSBio-2020) and **11** (LASSBio-2065), which were active in the assay. Compound **5b** (LASSBio-2020) displayed IC_50_ values of 13 and 7.1 μM for ROCK1 and ROCK2, respectively, whereas the *N*-methylated analogue **11** (LASSBio-2065) exhibited IC_50_ values of 3.1 and 3.8 μM for ROCK1 and ROCK2, respectively. The *N*-methylation introduced in compound **11** (LASSBio-2065) resulted in a slight increase in inhibitory potency, with a decrease in the IC_50_ of approximately 4-fold for ROCK1 and 1.8-fold for ROCK2.

Furthermore, considering that **2** (LASSBio-1524) was used in the structural design of this new family of ROCK inhibitors we also investigated the ability of **5b** (LASSBio-2020) and **11** (LASSBio-2065) to inhibit IKK-β. However, none of these *N*-sulphonylhydrazone derivatives was able to inhibit IKK-β at the screening concentration of 10 *μ*M, indicating their selectivity for the target kinases (data not shown).

Among the observed binding modes for compounds **5b** (LASSBio-2020) and **11** (LASSBio-2065), all docking conformations reproduced the key interaction with the hinge region through a hydrogen bond between the lone pair of the isoquinoline ring and a hydrogen of the amide backbone of MET156, which was essential for kinase molecular recognition (Figure 7). Both compounds adopt similar orientations in the active site, forming hydrophobic interactions with PHE368, ILE80, ALA215 and VAL90. However, we also considered the differences between the binding modes of compounds **5b** (LASSBio-2020) and **11** (LASSBio-2065) that could explain the bioactivity profiles observed in the present study. Apparently, the rotation of the N-S bond provided by the *N*-methyl group of compound **11** (LASSBio-2065) allows the compound to participate in an additional hydrophobic interaction with LEU205, which was not observed for the analogue **5b** (LASSBio-2020) and could explain the subtle difference in the potency of these compounds.

Nevertheless, this structural modification had no effect on ROCK1 and ROCK2 selectivity. Among the imine substituents chosen, the phenyl group was the most adequate for molecular recognition in ROCK. A likely explanation may be that the chosen 4-substituted derivatives apparently did not fit well in the catalytic cavity due to stereo-electronic effects.

#### Cytotoxic studies

**Figure 8.**
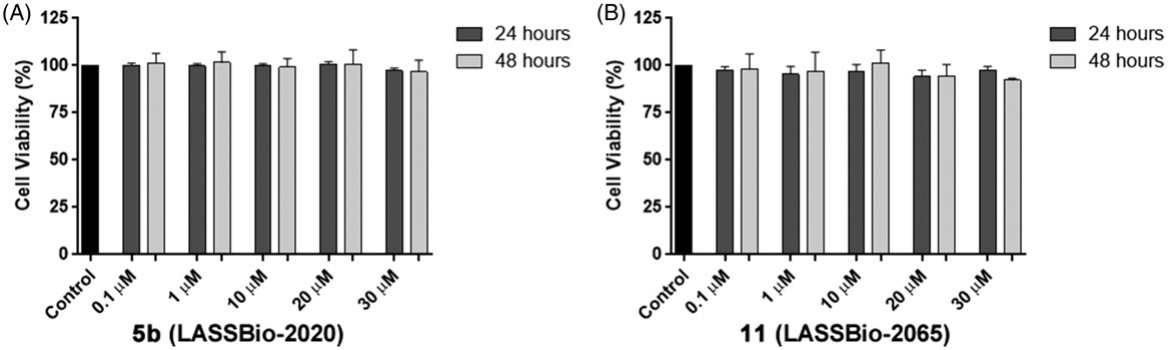
(A) Viability of MDA-MB-231 cells in the presence of compounds **5b** (LASSBio-2020) and (B) **11** (LASSBio-2065).

In the development of new drug candidates, concerns regarding cytotoxic and pharmacokinetic properties in early stages are important to address to avoid problems that require new steps of prototype optimization. The compounds were evaluated to determine their potential cytotoxic activities. All compounds were submitted to the MDA-MB-231 cell line viability assay using the MTT quantitative colorimetric method^15^. Relative cell viability was expressed as a percentage relative to control, untreated cells, and none of the evaluated compounds showed toxicity at the tested concentrations at 24 and 48 h. As an example, Figure 8 shows the cell viability at concentrations of compounds **5b** (LASSBio-2020) and **11** (LASSBio-2065) ranging from 0.1 to 30 μM.

**Figure 9.**
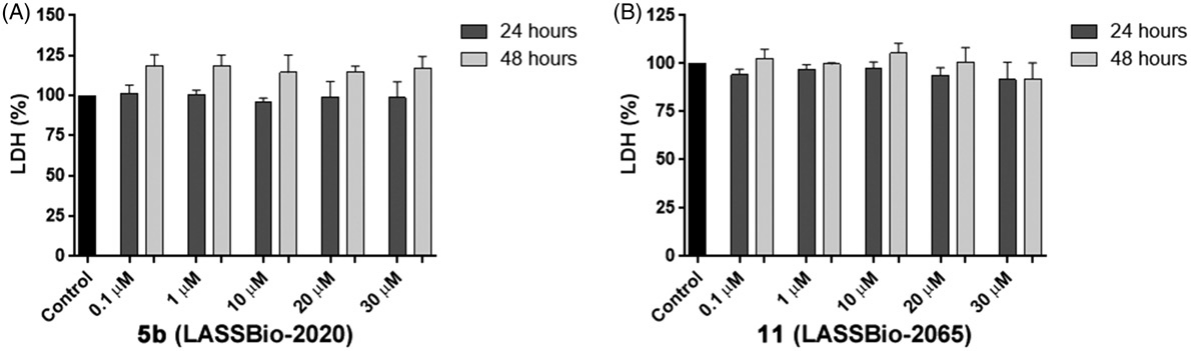
Measurements the serum lactate dehydrogenase (LDH) levels in the culture medium of MDA-MB-231 cells treated with compounds **5b** (LASSBio-2020) (A) and **11** (LASSBio- 2065) (B).

We also analysed the plasma membrane integrity by measuring the serum lactate dehydrogenase (LDH) levels in the culture medium to confirm the cell viability data. At concentrations of compounds **5b** (LASSBio-2020) and **11** (LASSBio-2065) ranging from 0.1 to 30 µM, LDH levels in the MDA-MB 231 cells were not altered after 24 and 48 h of incubation (Figure 9).

#### Cell-based scratch assay

The functions of Rho-kinase include the regulation of cellular contraction, motility, morphology, polarity, division, and gene expression^40^. Therefore, we performed a cell-based scratch assay followed by the quantification of the wounded area to analyse whether compounds **5b** (LASSBio-2020) and **11** (LASSBio-2065) altered cell migration.

**Figure 10.**
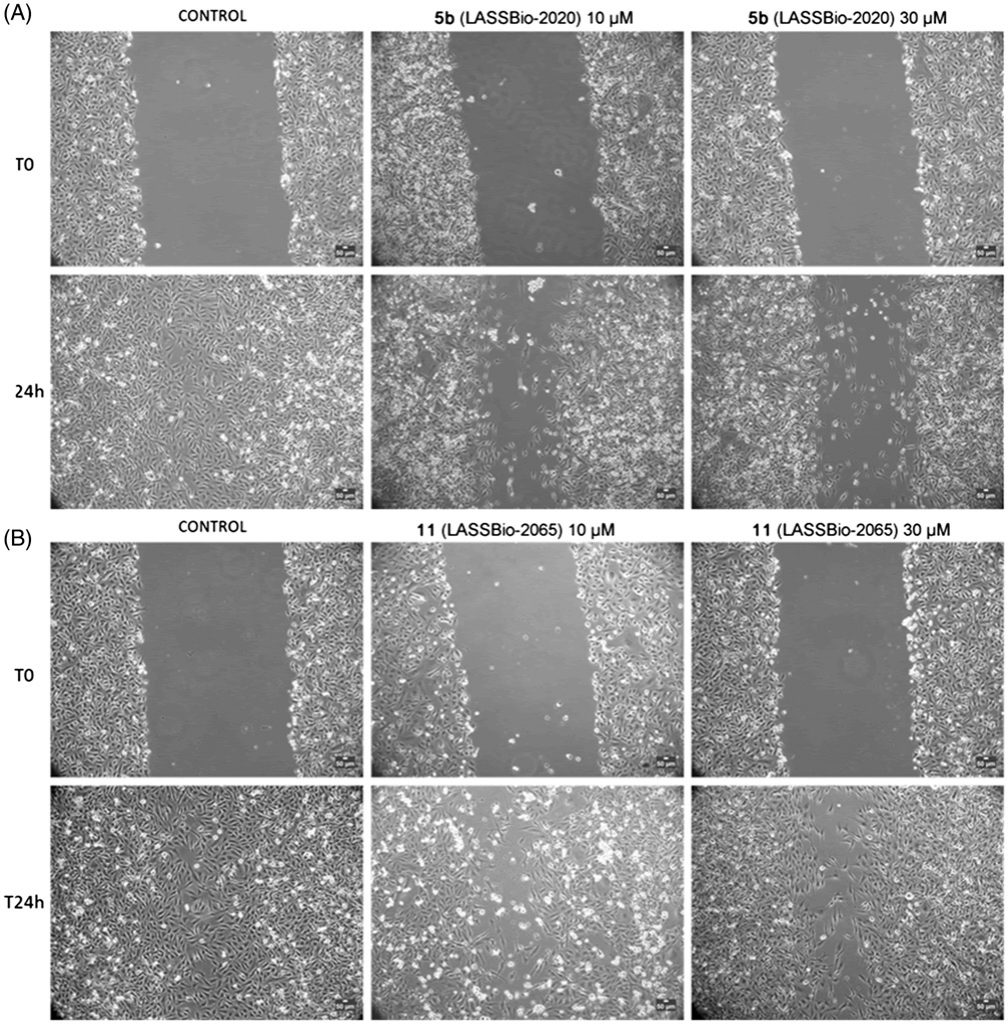
(A) Effects of compounds **5b** (LASSBio-2020) and (B) **11** (LASSBio-2065) on the migration of MDA-MB 231 cells. Images were obtained using phase-contrast microscopy. Filled areas represented migrating cells were calculated using ImageJ software. Scale bars represent 50 μm.

**Figure 11.**
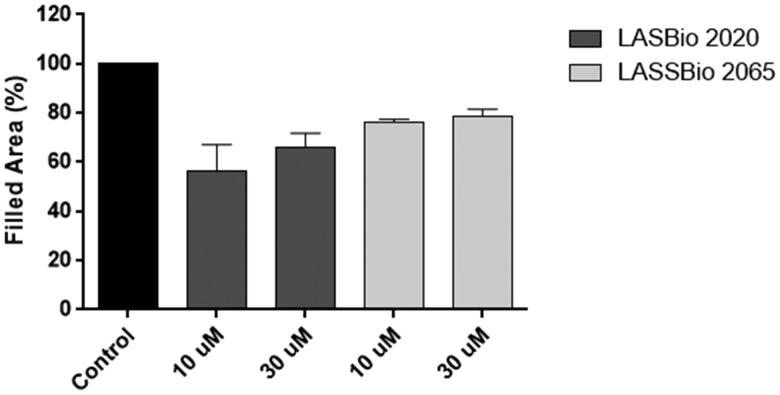
Effects of compounds **5b** (LASSBio-2020) and **11** (LASSBio-2065) on the migration of MDA-MB 231 cells. Filled areas representing migrating cells were calculated using ImageJ software. The results are presented as the medians ± standard deviations (*n* = 3) of independent experiments. Statistical analyses were performed using analysis of variance followed by the Newman-Keuls post-test. *p < 0.05 compared with the control group.

The cells were treated with compounds **5b** (LASSBio-2020) or **11** (LASSBio-2065) (10 or 30 μM) for 24 h. Interestingly, untreated cells nearly completely covered the scratched areas of the dish in 24 h, whereas in compound **5b-**treated cultures, relatively empty areas were visible in the culture dishes after 24 h of treatment with all tested concentrations compared to control (Figure 10). Treatment with compound **11** (LASSBio-2065) inhibited cell migration in a less pronounced manner than compound **5b** (LASSBio-2020). Empty areas ranged from 21% to 43% compared to control untreated cultures (Figure 11). Therefore, as we observed the complete coverage of the scratched areas of the dish in control cells after 24 h of incubation, both compounds, **5b** (LASSBio-2020) and **11** (LASSBio-2065), inhibited MDA-MB 231 cell migration. When comparing with data previously obtained by our group showing that fasudil inhibited MDA-MB231 migration,^15^ 50 µM fasudil reduced cell migration by 50% in the present study. Meanwhile, compounds **5b** (LASSBio-2020) and **11** (LASSBio-2065) presented similar effects at lower concentrations (10 µM). Based on our data, these new analogues seem to be more potent than fasudil at inhibiting tumour cell migration.

#### Chemical stability assay

**Figure 12.**
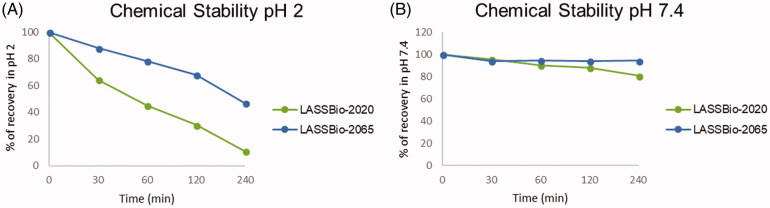
Chemical stability of compounds **5b** (LASSBio-2020) and **11** (LASSBio-2065). (A) Recovery (%) of compounds at pH 2. (B) Recovery (%) of compounds at pH 7.4. The experiments were conducted in triplicate, and the values represent the averages from the experiments.

Due to the possible chemical instability of the imine functional group present in the *N*-sulphonylhydrazone framework, the stability profiles of compounds **5b** (LASSBio-2020) and **11** (LASSBio-2065) were determined. The study was conducted under two conditions, pH 2.0 and pH 7.4, to mimic the acidic stomach and the neutral plasma environments, respectively. The compounds were examined in the presence of acid (pH 2) and neutral (pH 7.4) buffers with stirring for 240 min at a controlled temperature (37 °C). The results are shown in Figure 12.

Both compounds were stable in neutral medium (pH 7.4) and less than 20% degradation occurred, as observed from the compound recovery percentage after 4 h. However, under acidic conditions, both compounds underwent partial hydrolysis. After 30 min of incubation, the recovery percentage of compound **5b** (LASSBio-2020) was 64% compared to 88% for compound **11** (LASSBio-2065). This behaviour was progressively observed at 60, 120, and 240 min. The hydrolysis of imines are directly related to the electron density on the imine carbon. A lower electron density at this carbon indicates that this atom is more electrophilic and available for nucleophilic attack by water. The explanation for the better recovery percentage, which means a better chemical stability, of compound **11** (LASSBio-2065) is related to the presence of an additional methyl group that increases the electron density at the imine carbon and renders it less susceptible to hydrolysis than compound **5b** (LASSBio-2020).

## Conclusions

In the present study, we synthesized a new congener series of *N*-sulphonylhydrazones as ROCK inhibitors that were designed through molecular hybridization between the clinically approved drug fasudil (**1**) and the IKK-β inhibitor LASSBio-1524 (**2**). After an initial screen of their abilities to inhibit both ROCK1 and ROCK2 isoforms, compound **5b** (LASSBio-2020) was identified as a nonselective inhibitor of ROCK1 and ROCK2 that presented moderate potency. The corresponding NAH analogue **10** (LASSBio-2064) was inactive in the ROCK inhibitory assay, revealing the pharmacophoric nature of the sulphonyl group. On the other hand, the *N*-methylated analogue **11** (LASSBio-2065) was a slightly more potent ROCK inhibitor, but did not alter the selectivity profile between ROCK1 and ROCK2 isoforms. In cell-based assays, derivatives **5b** (LASSBio-2020) and **11** (LASSBio-2065) were also effective at inhibiting cell migration. Furthermore, none of the synthesized compounds were cytotoxic in LDH and MTT assays. This molecular scaffold showed similar inhibitory potency to the clinically approved drug fasudil (**1**) and can be utilized for the further optimization and development of ROCK inhibitors.

## Supplementary Material

Supplemental Material
