# Supplementary material for: Synthesis and pharmacological evaluation of novel isoquinoline N-sulphonylhydrazones designed as ROCK inhibitors
Source: J Enzyme Inhib Med Chem. 2018 Jul 25;33(1):1181–93. doi: 10.1080/14756366.2018.1490732 (PMC6060383; doi:10.1080/14756366.2018.1490732)
Supplement: Supplemental Material [file IENZ_A_1490732_SM0886.pdf]

_Supporting Information

Synthesis and Pharmacological Evaluation of Novel Isoquinoline *N*-Sulfonylhydrazones Designed as ROCK Inhibitors

Ramon Guerra de Oliveira,[a,b] Fabiana Sélos Guerra,[b,c] Cláudia dos Santos Mermelstein,[d] Patrícia Dias Fernandes,[b,c] Isadora Tairinne de Sena Bastos,[e] Fanny Nascimento Costa,[f] Regina Cely Rodrigues Barroso,[e] Fabio Furlan Ferreira,[f] Carlos A. M. Fraga[a,b,]*

[a]Laboratório de Avaliação e Síntese de Substâncias Bioativas (LASSBio®), Instituto de Ciências Biomédicas, Universidade Federal do Rio de Janeiro, PO Box 68023, 21941-902, Rio de Janeiro, RJ, Brasil.

[b]Programa de Pós-Graduação em Farmacologia e Química Medicinal, Instituto de Ciências Biomédicas, Universidade Federal do Rio de Janeiro, 21941-902, Rio de Janeiro, RJ, Brasil.

[c]Laboratório de Farmacologia da Dor e da Inflamação, Instituto de Ciências Biomédicas, Universidade Federal do Rio de Janeiro, Universidade Federal do Rio de Janeiro, 21941-902, Rio de Janeiro, RJ, Brasil.

[d]Laboratório de Diferenciação Muscular, Instituto de Ciências Biomédicas, Universidade Federal do Rio de Janeiro, Rio de Janeiro, Brasil.

[e]Physics Institute, UERJ, Rio de Janeiro-RJ 20550-900, Brasil

[f]Centro de Ciências Naturais e Humanas (CCNH), Universidade Federal do ABC(UFABC) Av. dos Estados 5001, Santo André-SP 09210-580, Brasil.

*Corresponding author. E-mail: cmfraga@ccsdecania.ufrj.br; Phone: +55 21 3938-6502

CONTENTS

1. NMR spectra of final compounds (**5a-h**, **10** and **11**)
2. HRMS of Final Compounds (**5a-h**, **10** and **11**)
3. Curves of Determination of the IC_{50} of the compounds (**5b**, **10** and **11**)
4. X-ray Diffraction Data of **5f**

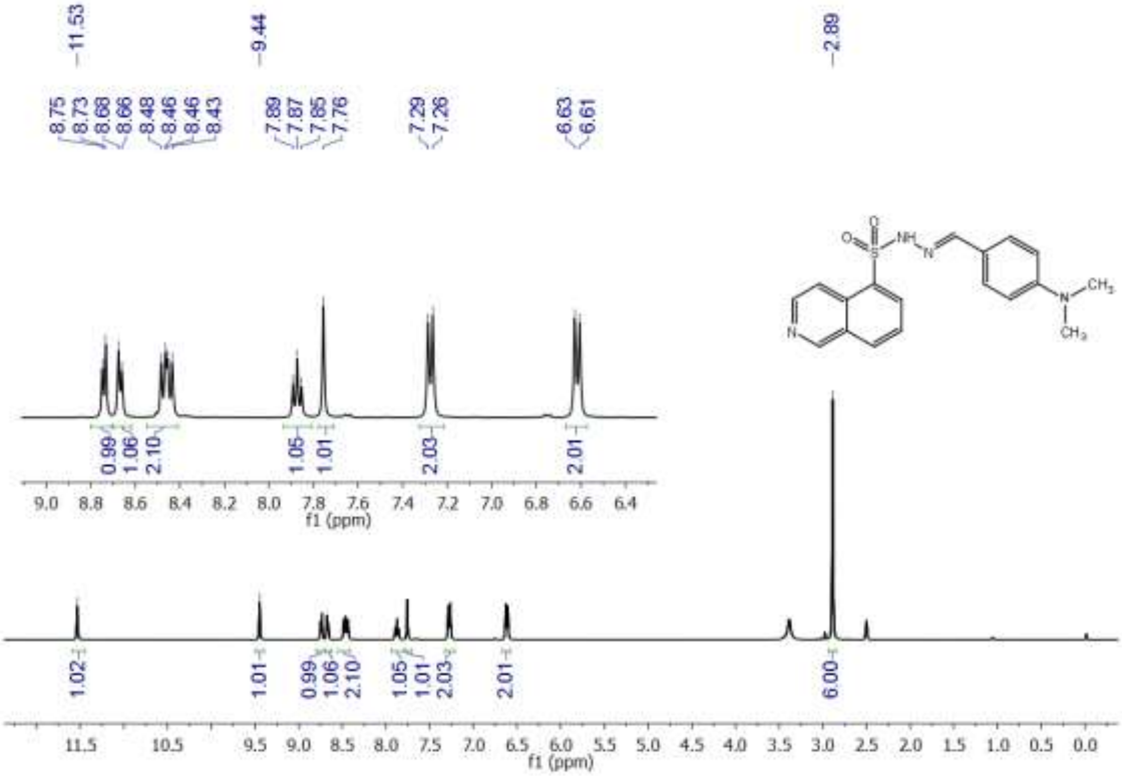

Figure 1 - Compound **5a** (^{1}H NMR, 400 MHz, DMSO-d6, TMS).

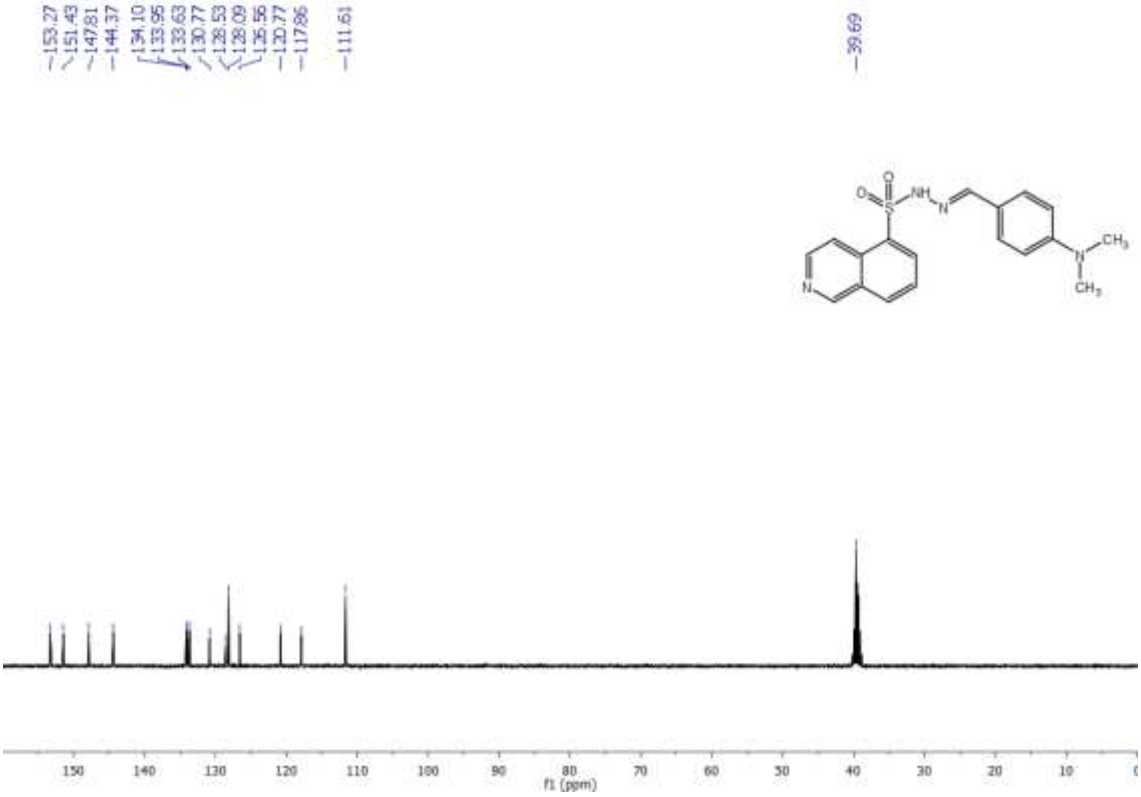

Figure 2 - Compound **5a** (^{13}C NMR, 100 MHz, DMSO-d6, TMS).

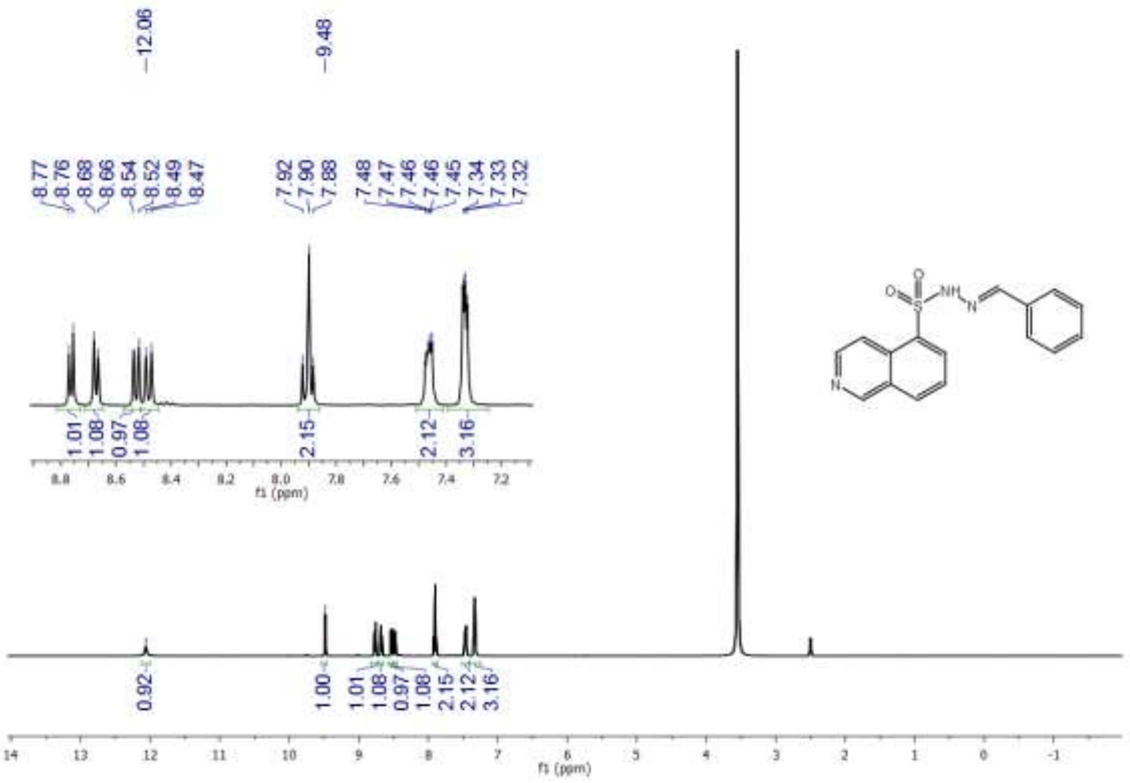

Figure 3 - Compound **5b** (^{1}H NMR, 400 MHz, DMSO-d6, TMS).

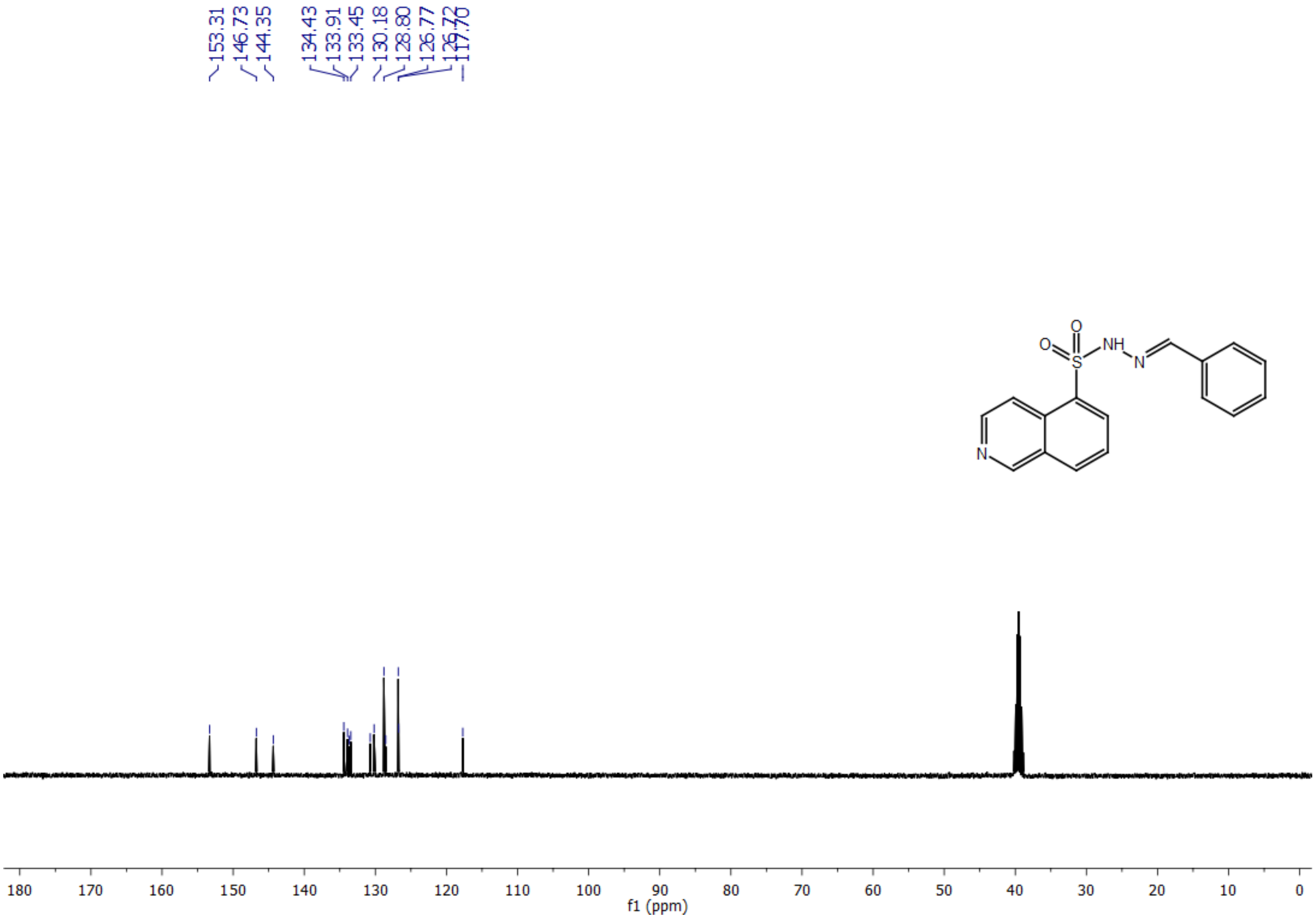

Figure 4 - Compound **5b** (^{13}C NMR, 100 MHz, DMSO-d6, TMS).

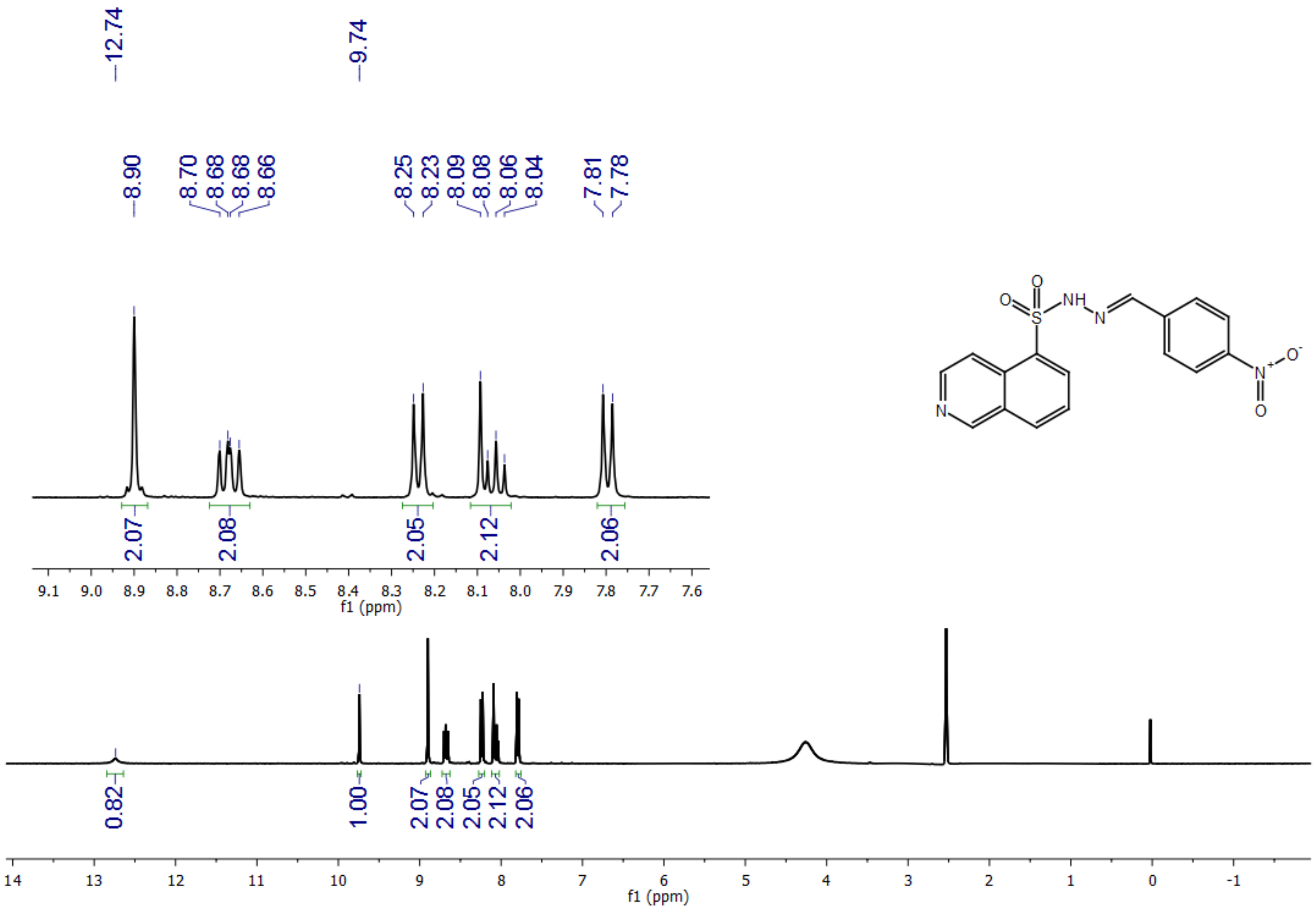

Figure 5 - Compound **5c** (^{1}H NMR, 400 MHz, DMSO-d6, TMS).

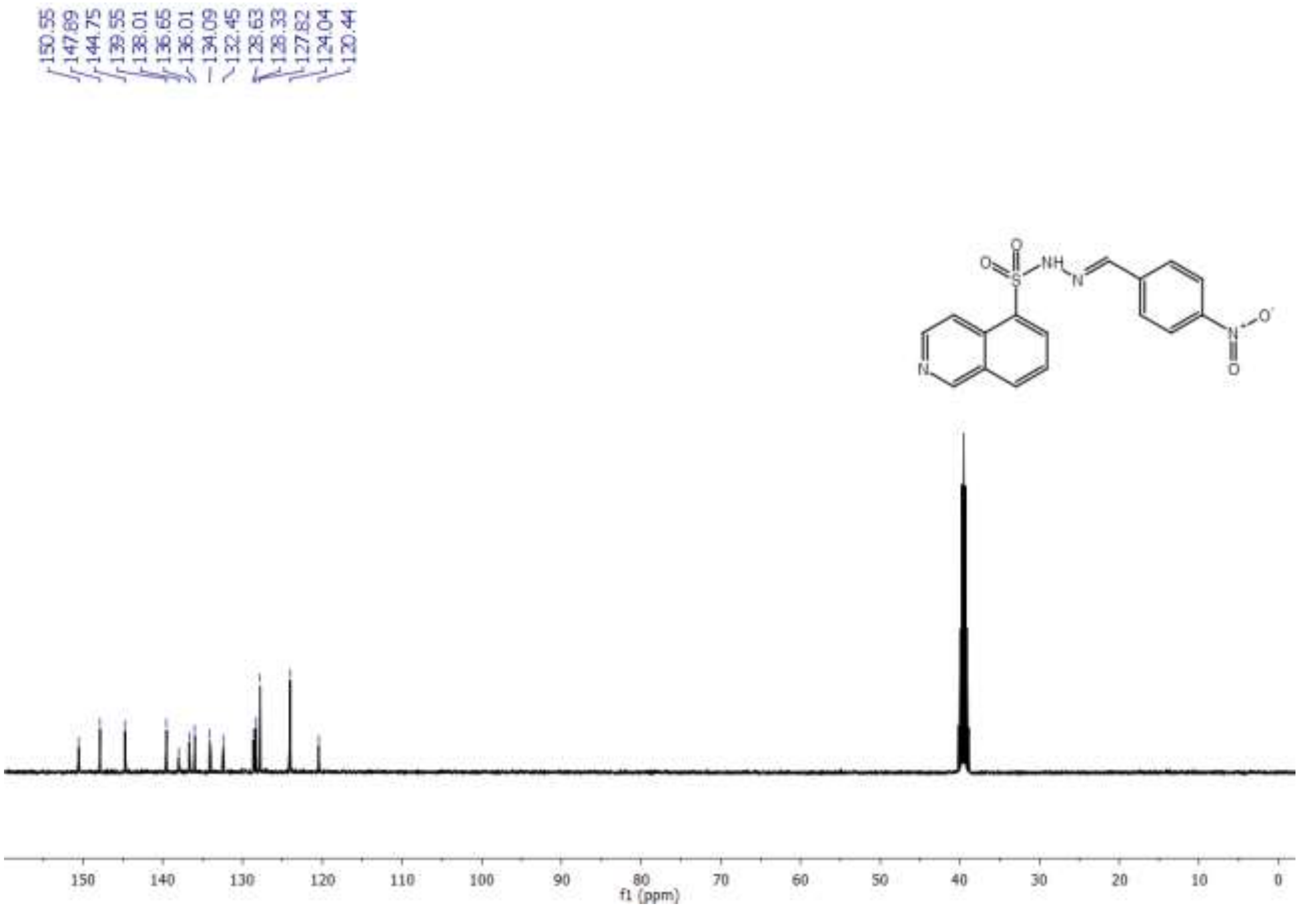

Figure 6 - Compound **5c** (^{13}C NMR, 100 MHz, DMSO-d6, TMS).

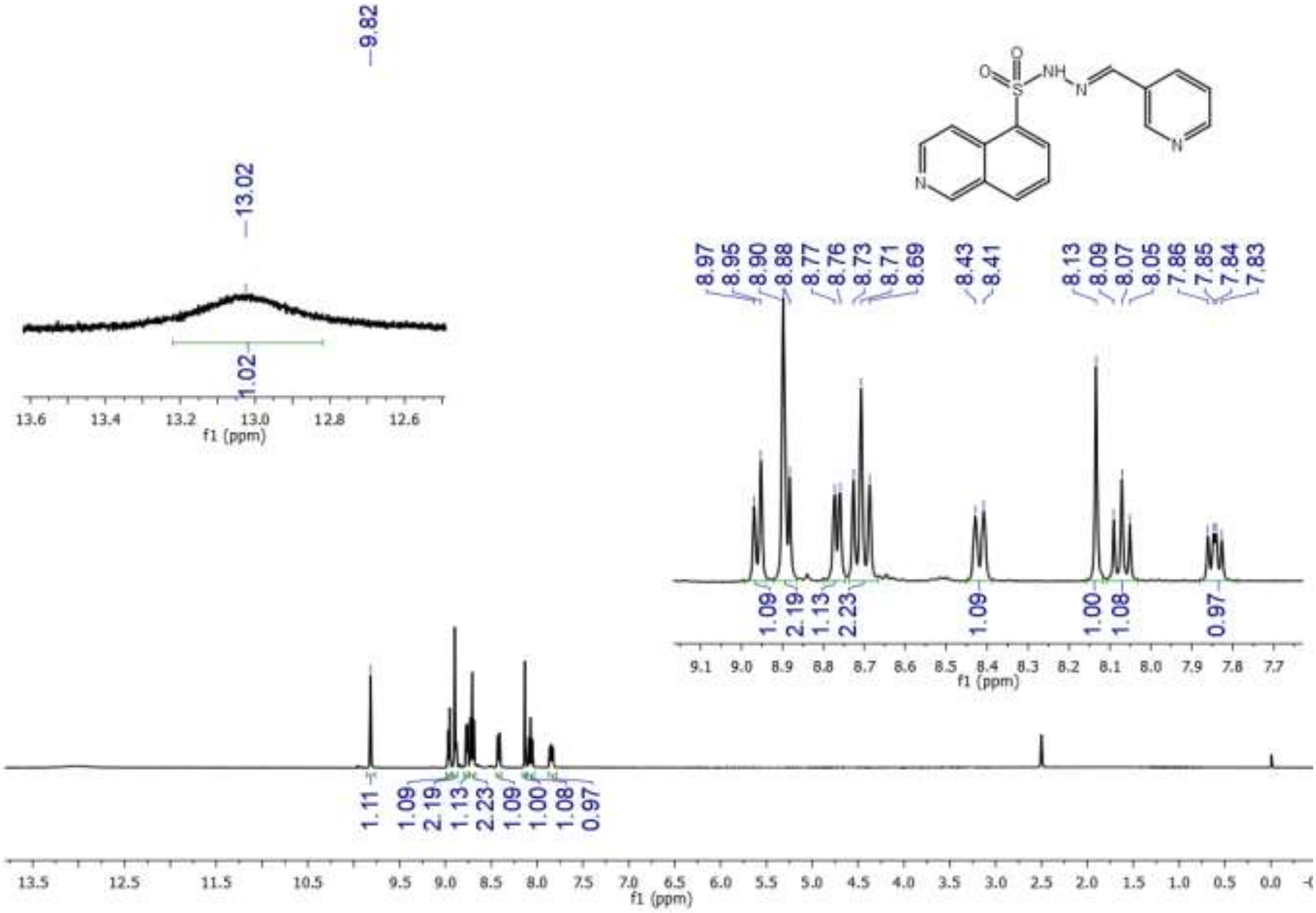

Figure 7 - Compound **5d** (^{1}H NMR, 400 MHz, DMSO-d6, TMS).

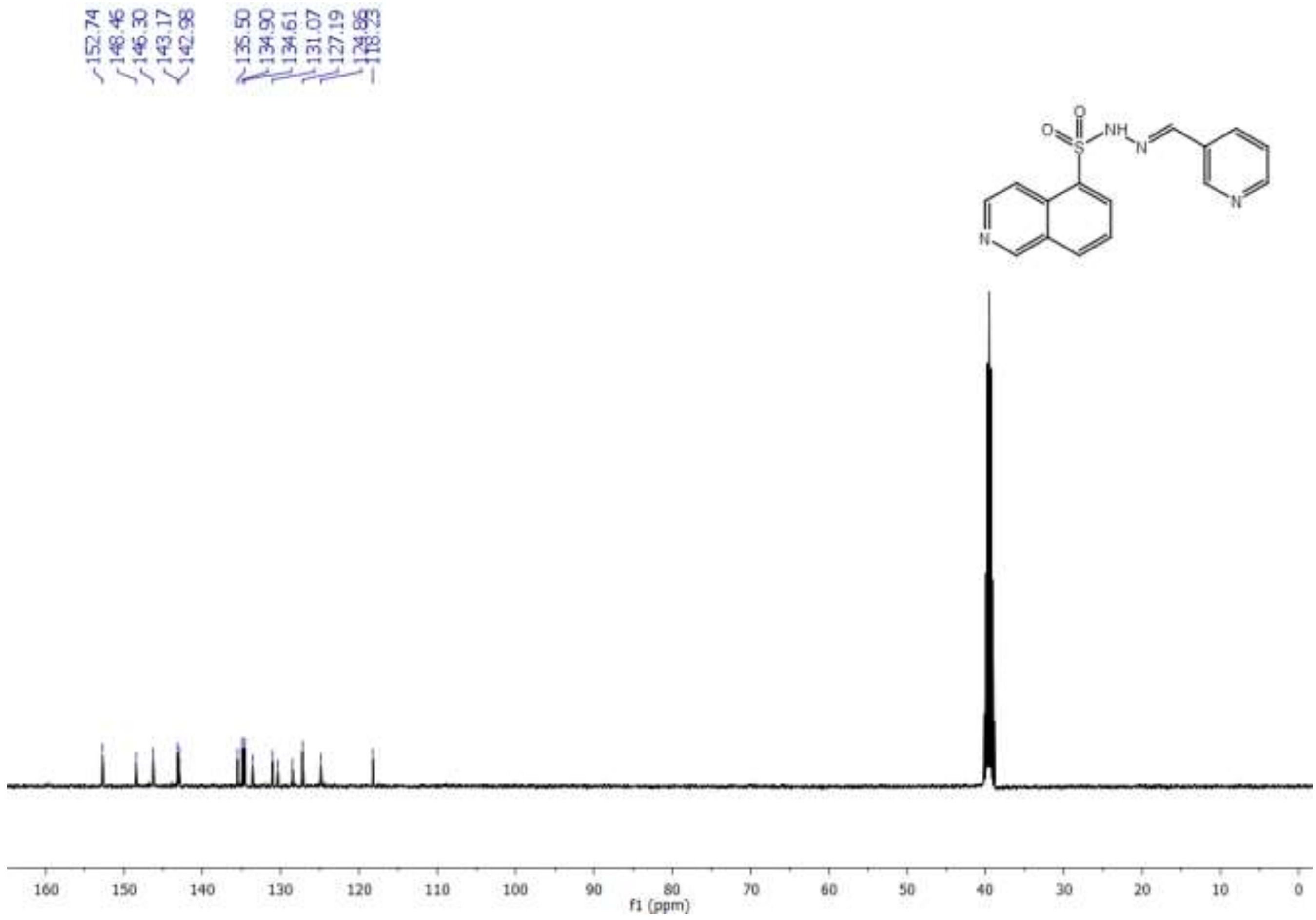

Figure 8 - Compound **5d** (^{13}C NMR, 100 MHz, DMSO-d6, TMS).

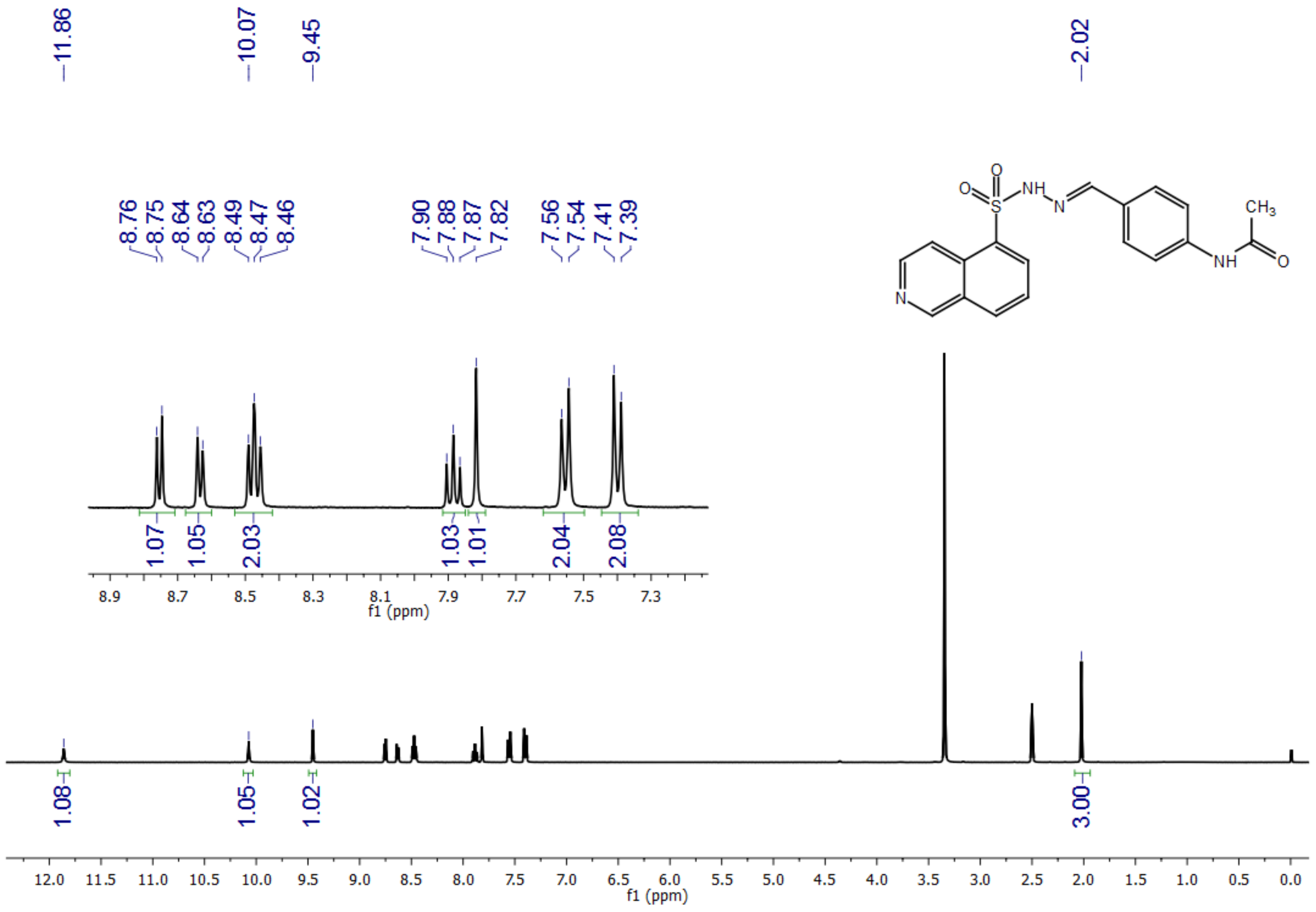

Figure 9 - Compound **5e** (^{1}H NMR, 400 MHz, DMSO-d6, TMS).

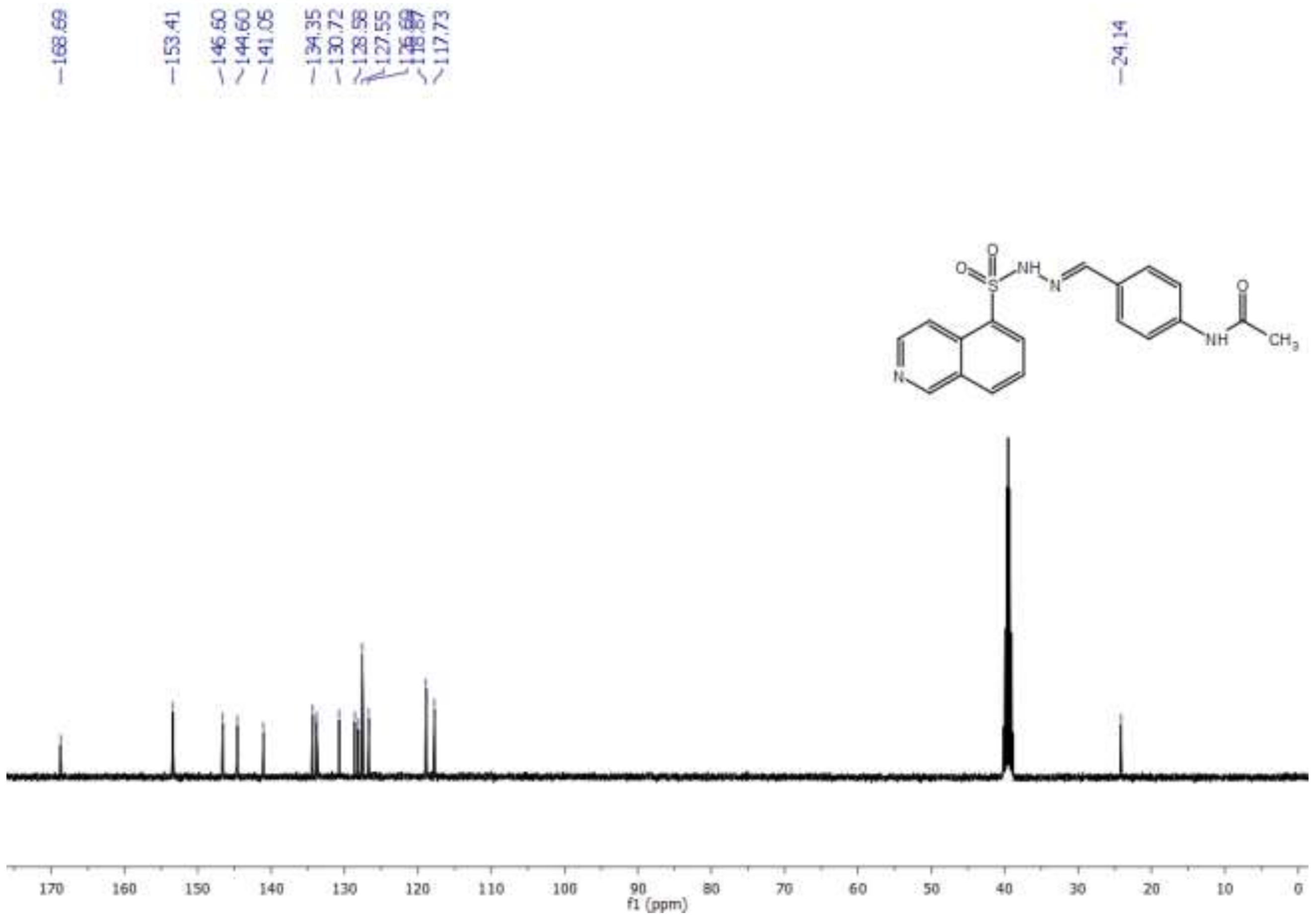

Figure 10 - Compound **5e** (^{13}C NMR, 100 MHz, DMSO-d6, TMS).

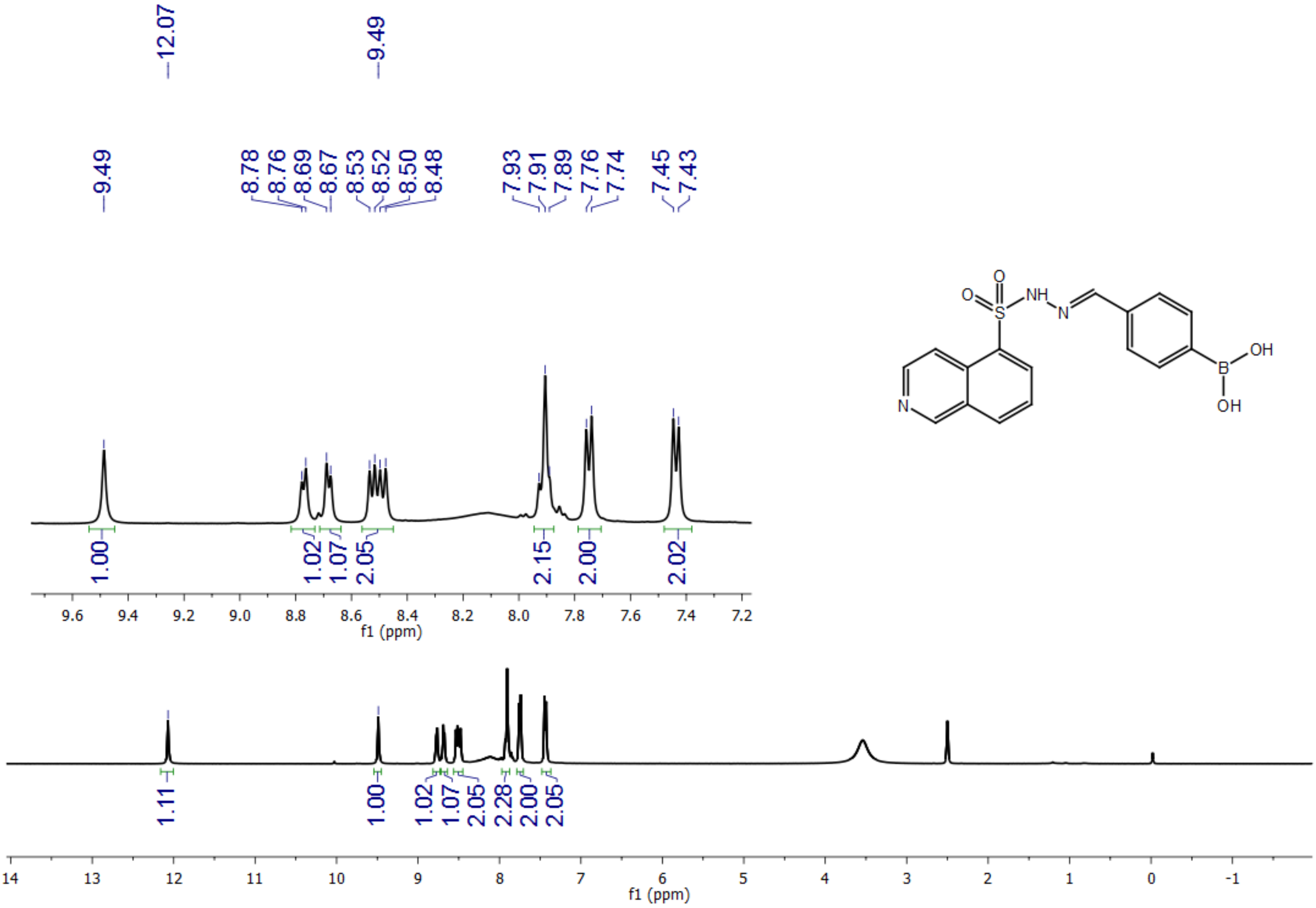

Figure 11 - Compound **5f** (^{1}H NMR, 400 MHz, DMSO-d6, TMS).

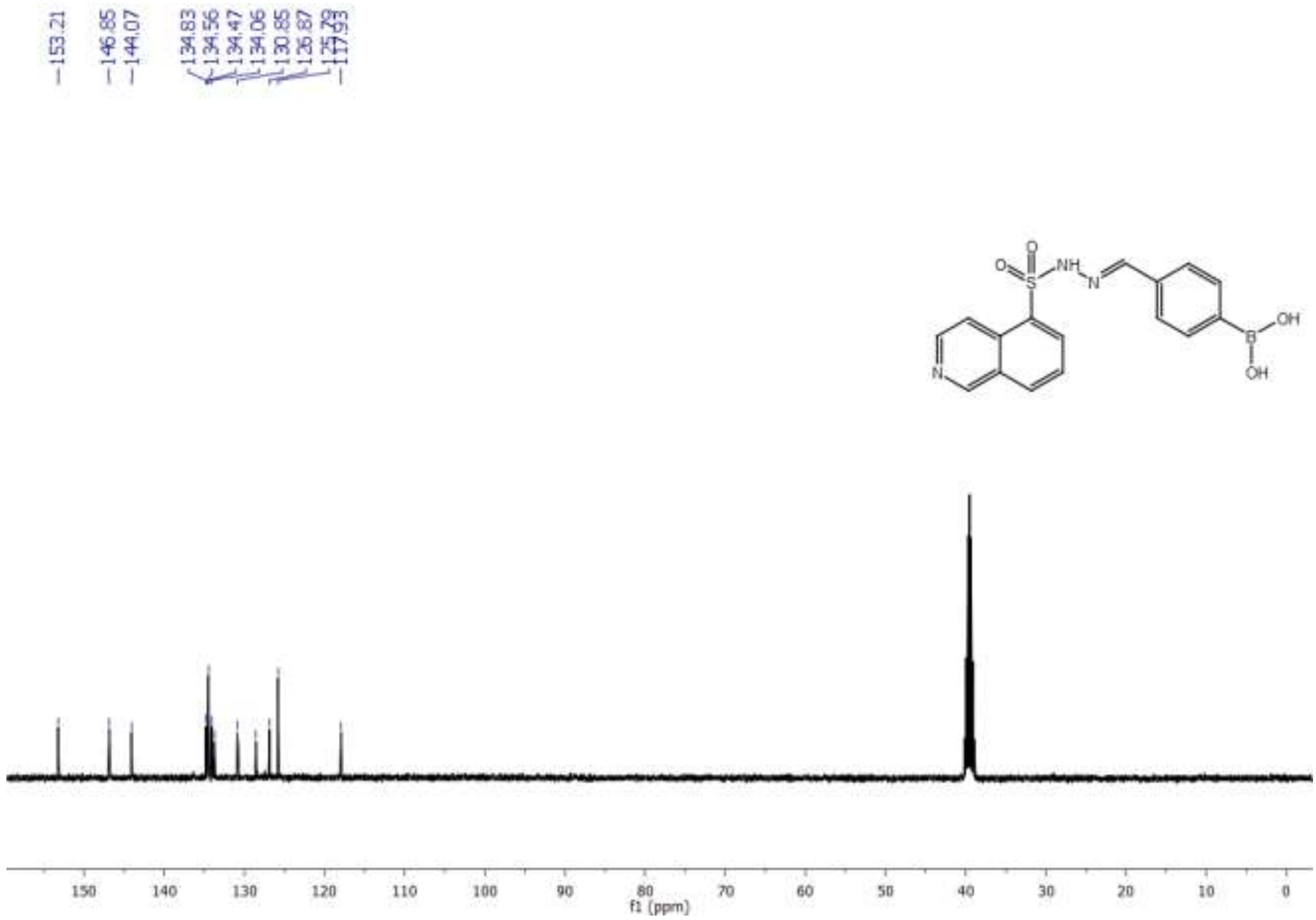

Figure 12 - Compound **5f** (^{13}C NMR, 100 MHz, DMSO-d6, TMS).

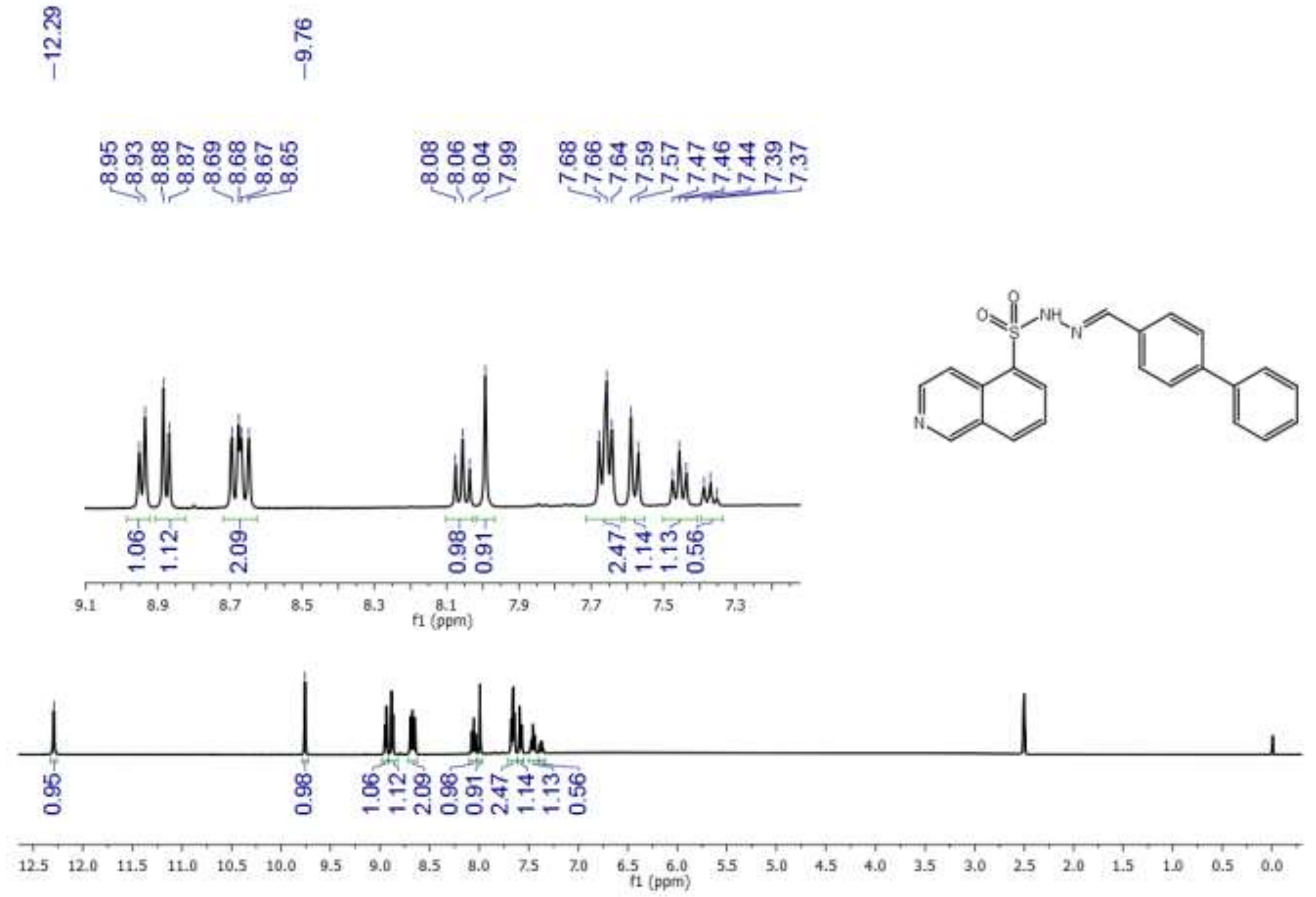

Figure 13 - Compound **5g** (^{1}H NMR, 400 MHz, DMSO-d6, TMS).

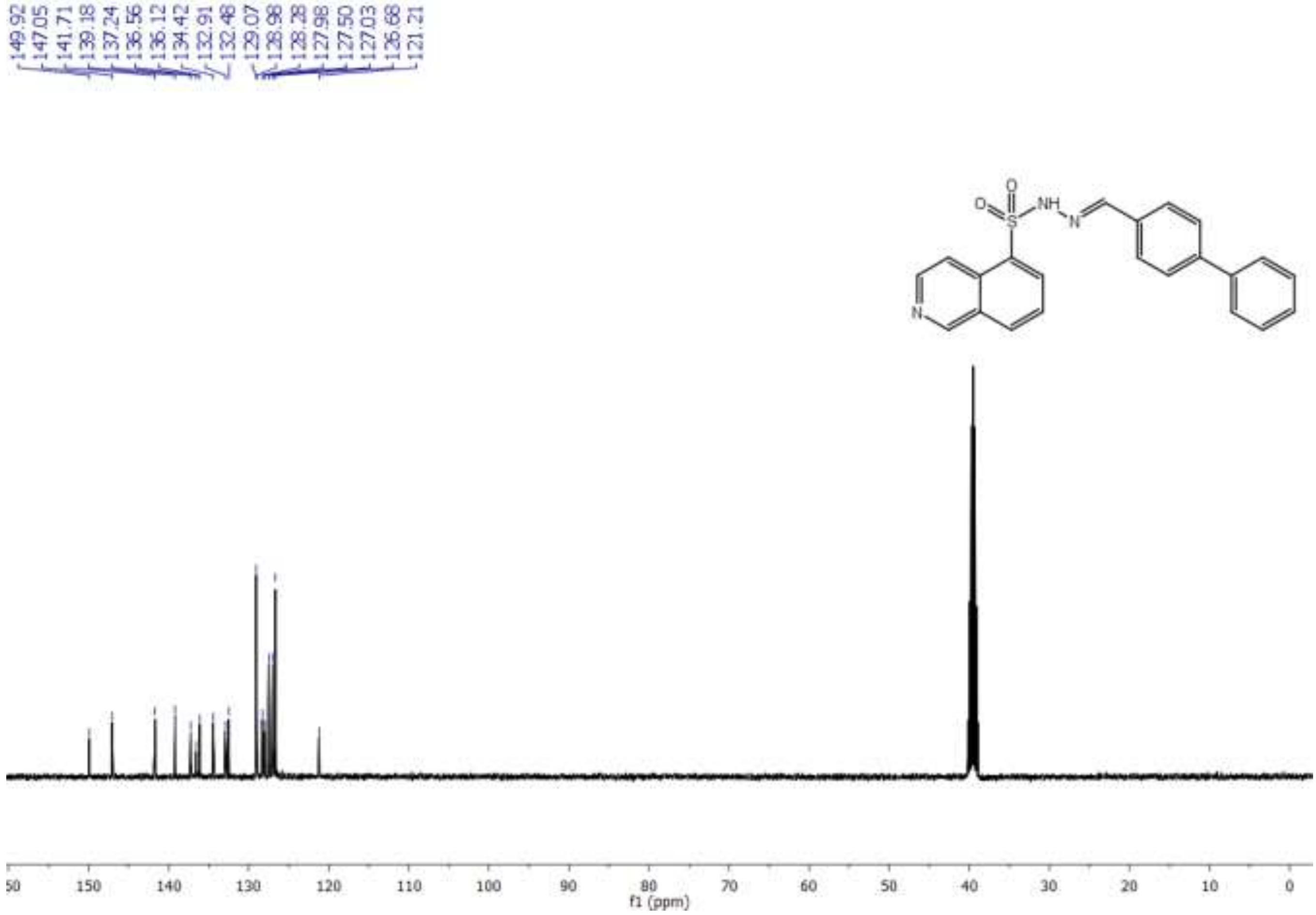

Figure 14 - Compound **5g** (^{13}C NMR, 100 MHz, DMSO-d6, TMS).

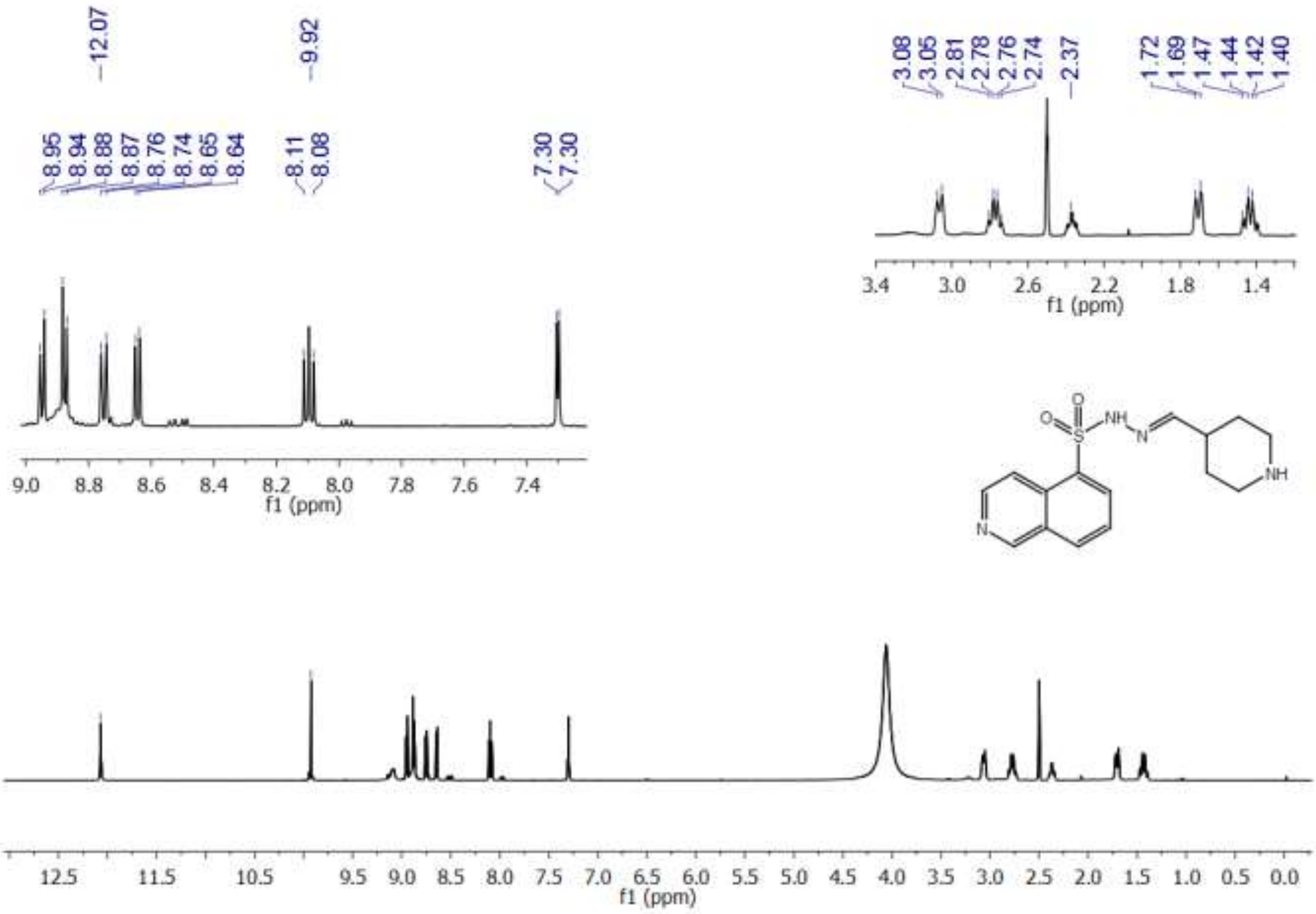

Figure 15 - Compound **5h** (^{1}H NMR, 400 MHz, DMSO-d6, TMS).

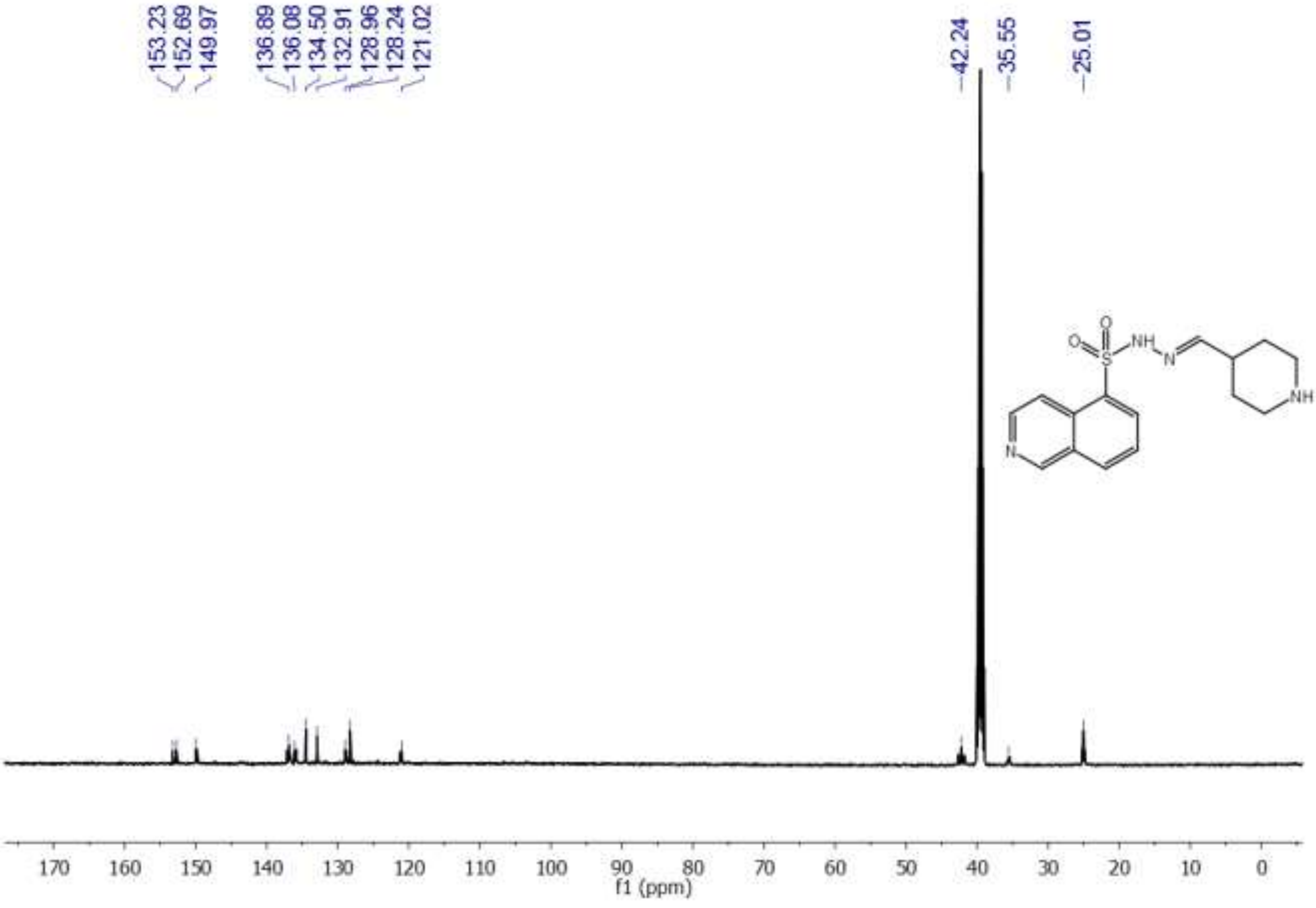

Figure 16 - Compound **5h** (^{13}C NMR, 100 MHz, DMSO-d6, TMS).

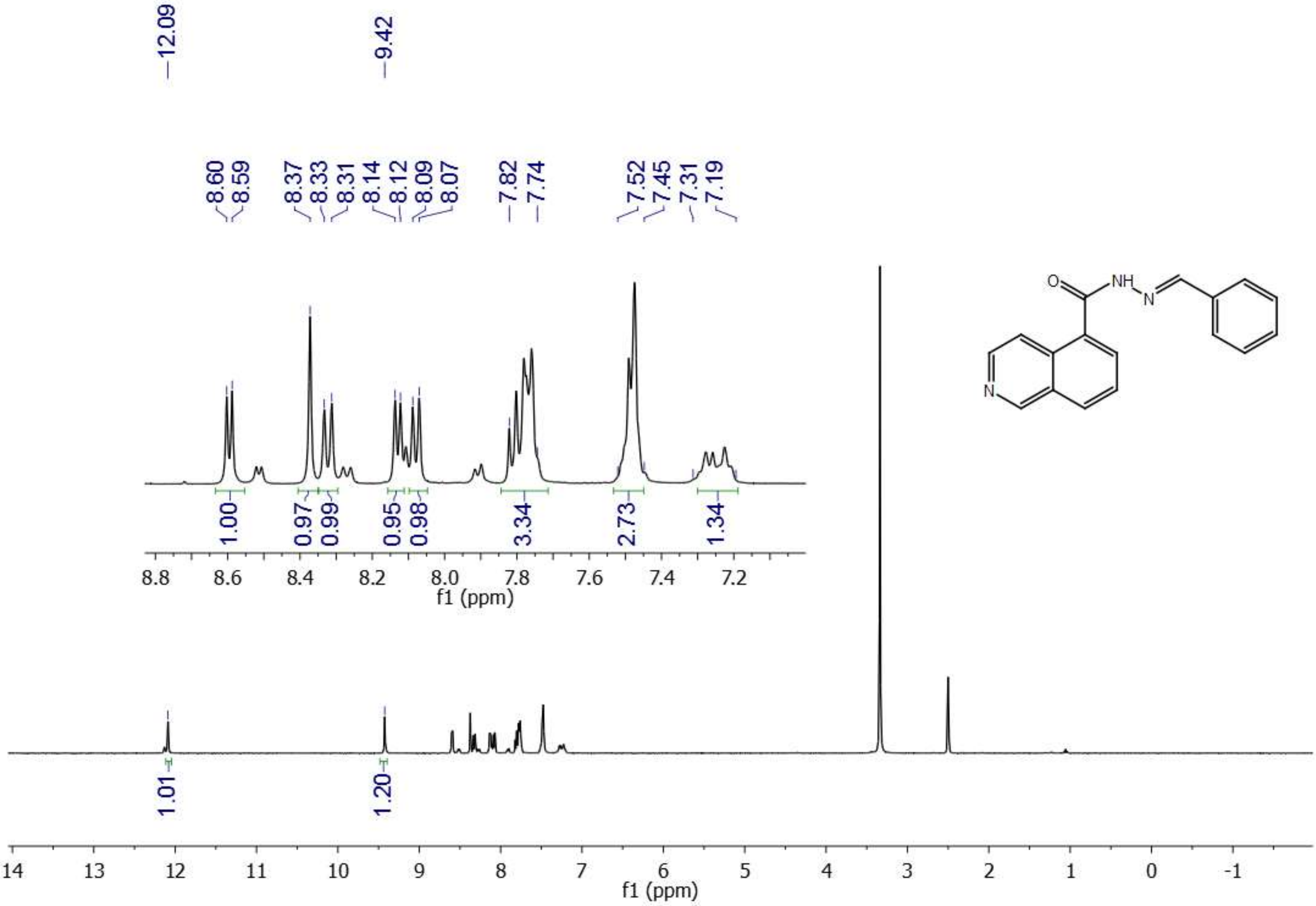

Figure 17 - Compound **10** (^{1}H NMR, 400 MHz, DMSO-d6, TMS).

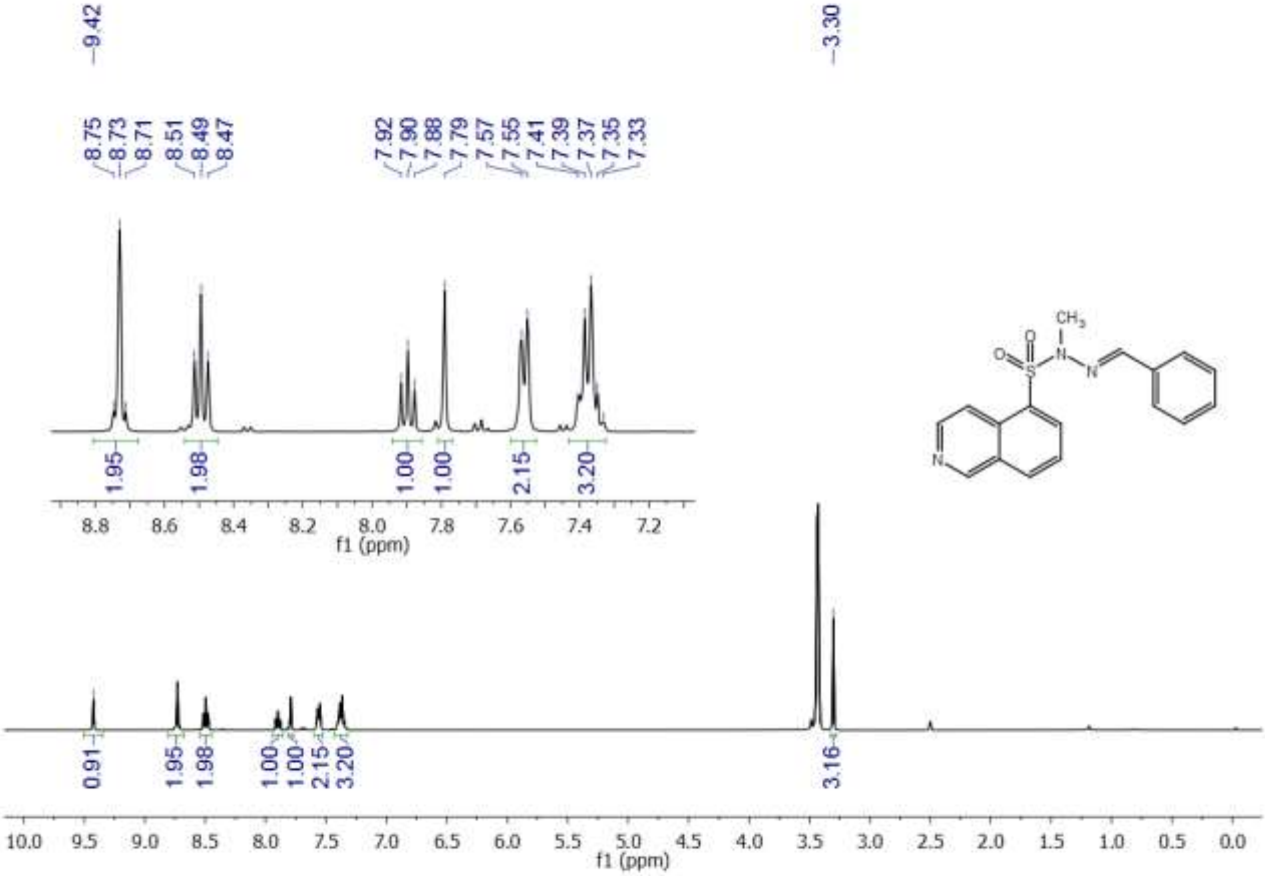

Figure 18 - Compound **11** (^{1}H NMR, 400 MHz, DMSO-d6, TMS).

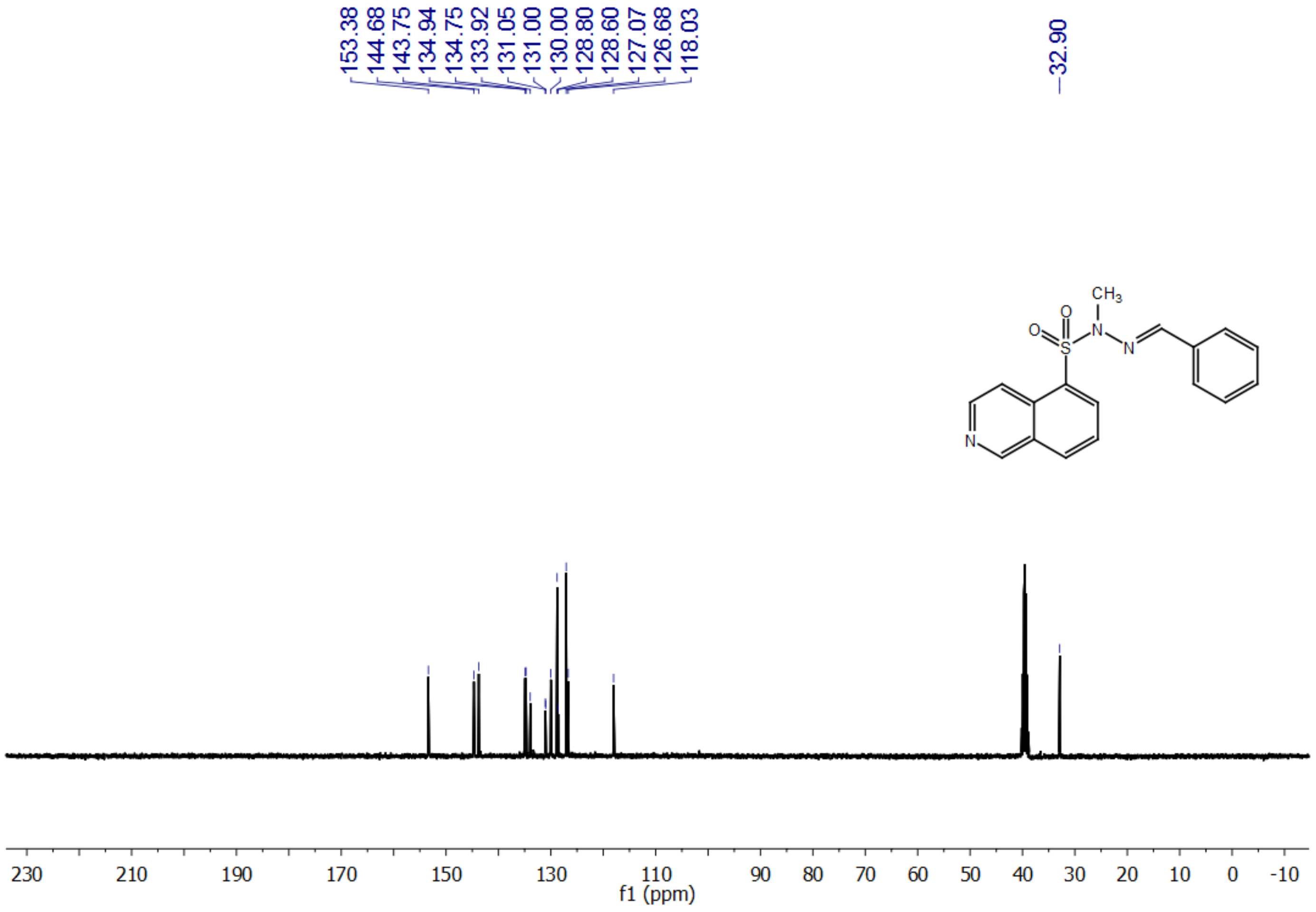

Figure 19 - Compound **11** (^{13}C NMR, 100 MHz, DMSO-d6, TMS).

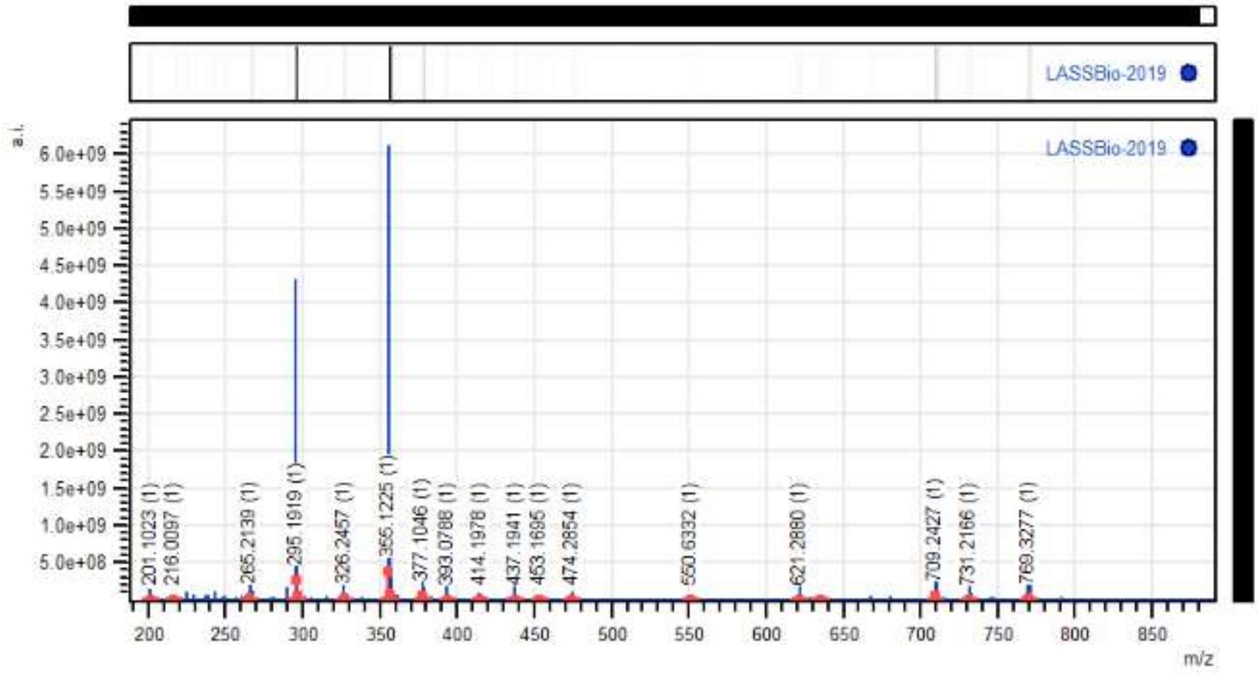

Figure 20– HRMS of **5a**(ESI-FT-ICR) ([M+H]$^+$).

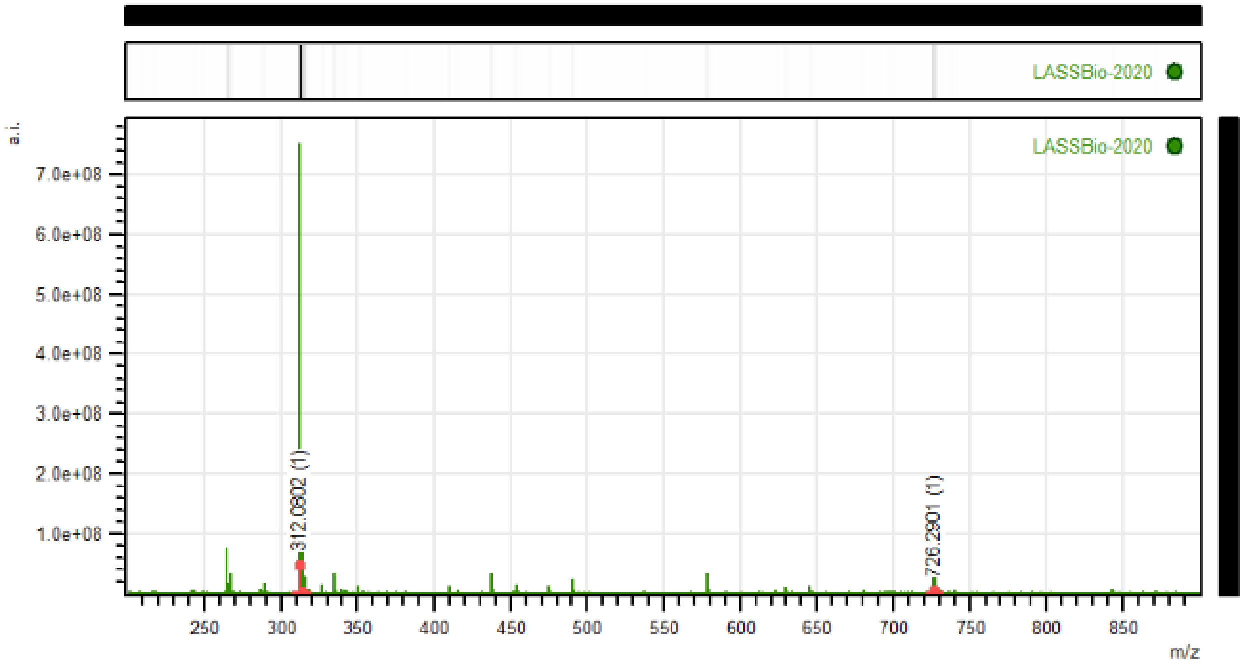

Figure 21 - HRMS of **5b** (ESI-FT-ICR) ($[M+H]^+$).

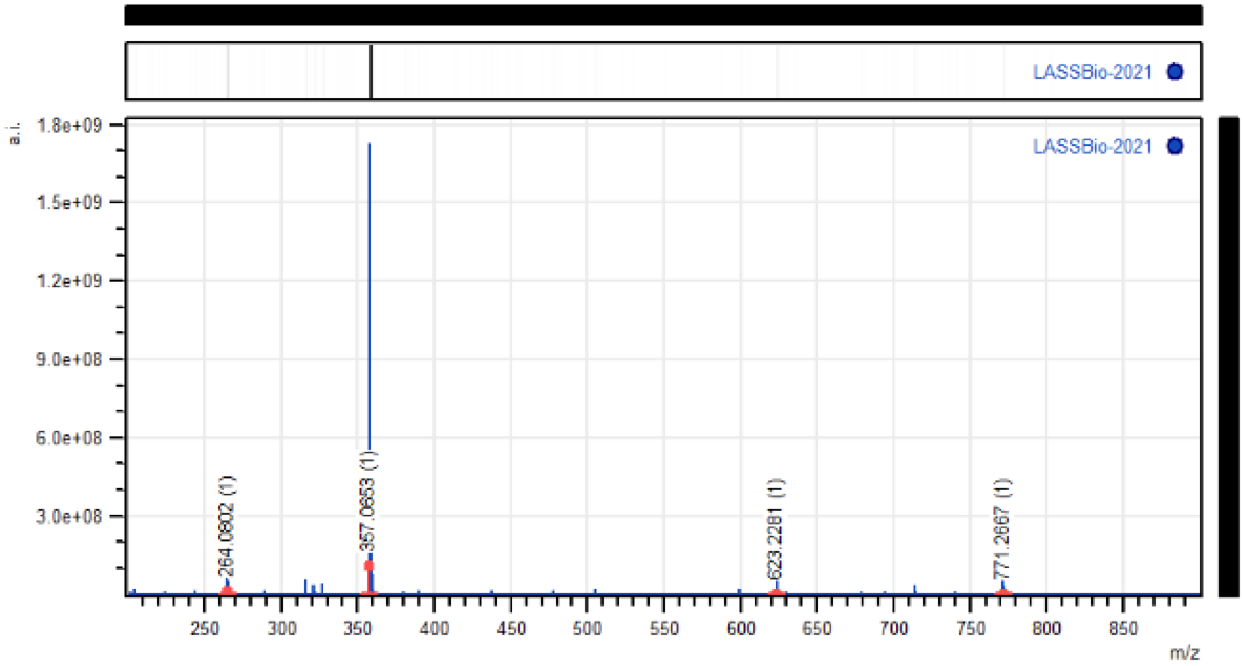

Figure 22 - HRMS of **5c** (ESI-FT-ICR) ($[M+H]^+$).

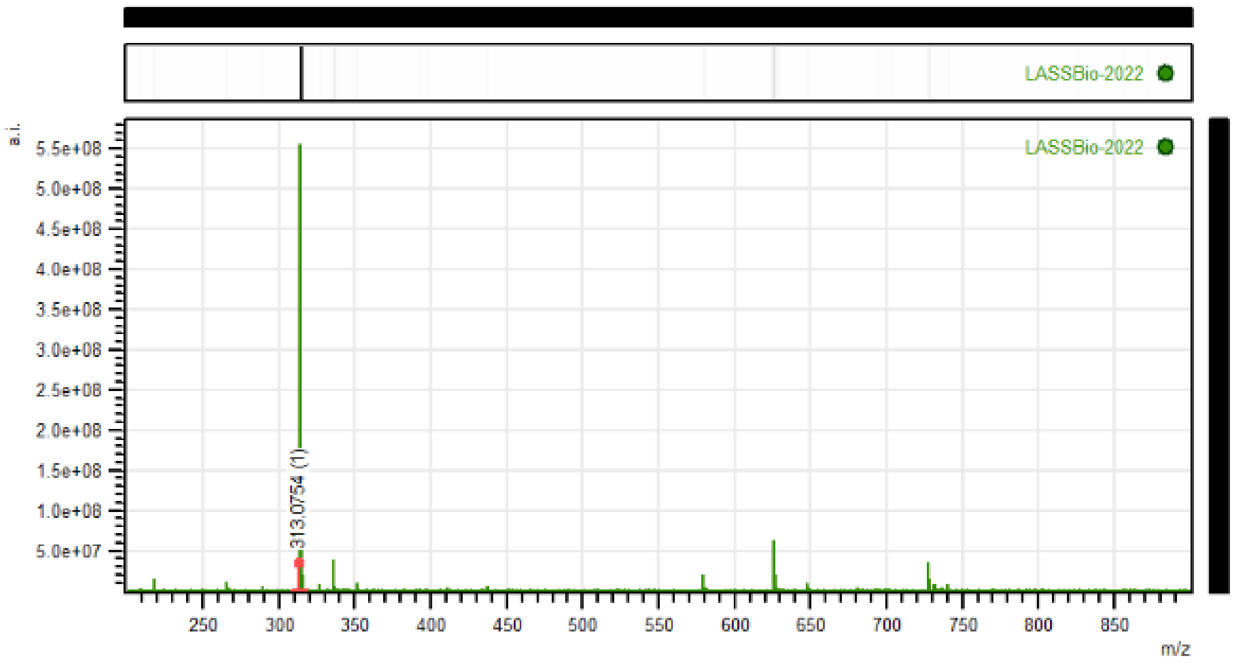

Figure 23 - HRMS of **5d** (ESI-FT-ICR) ([M+H]$^+$).

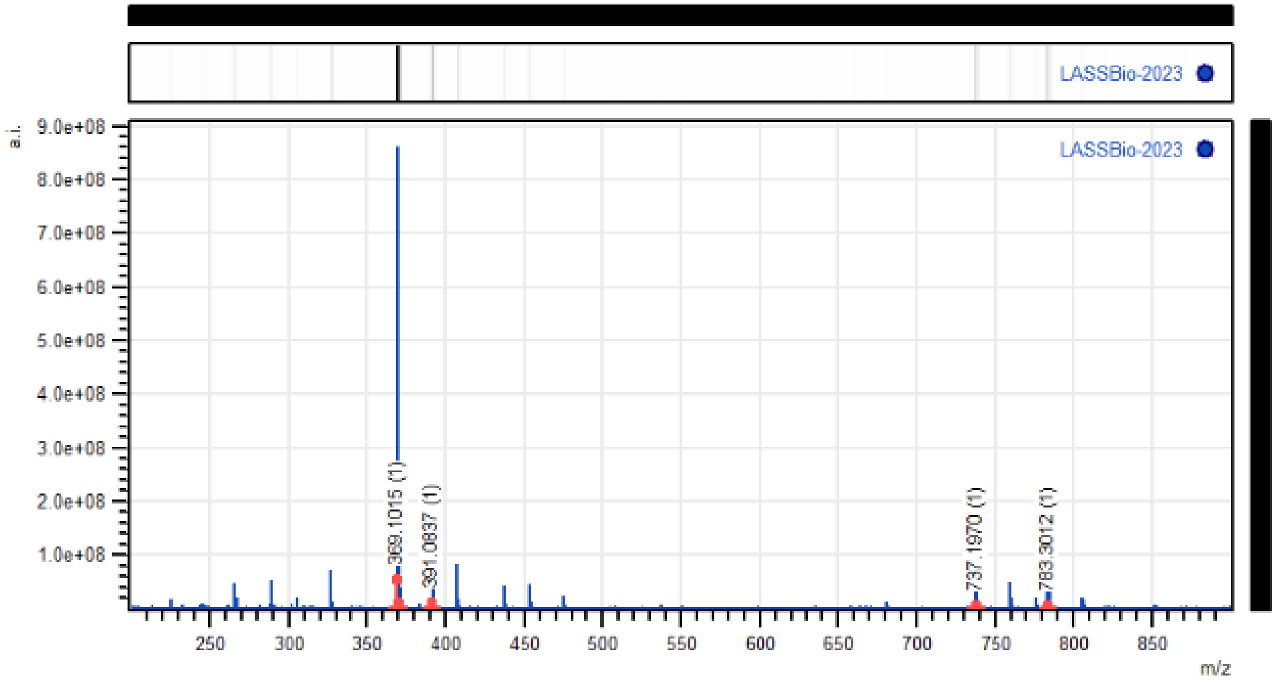

Figure 24 - HRMS of **5e** (ESI-FT-ICR) ([M+H]$^+$).

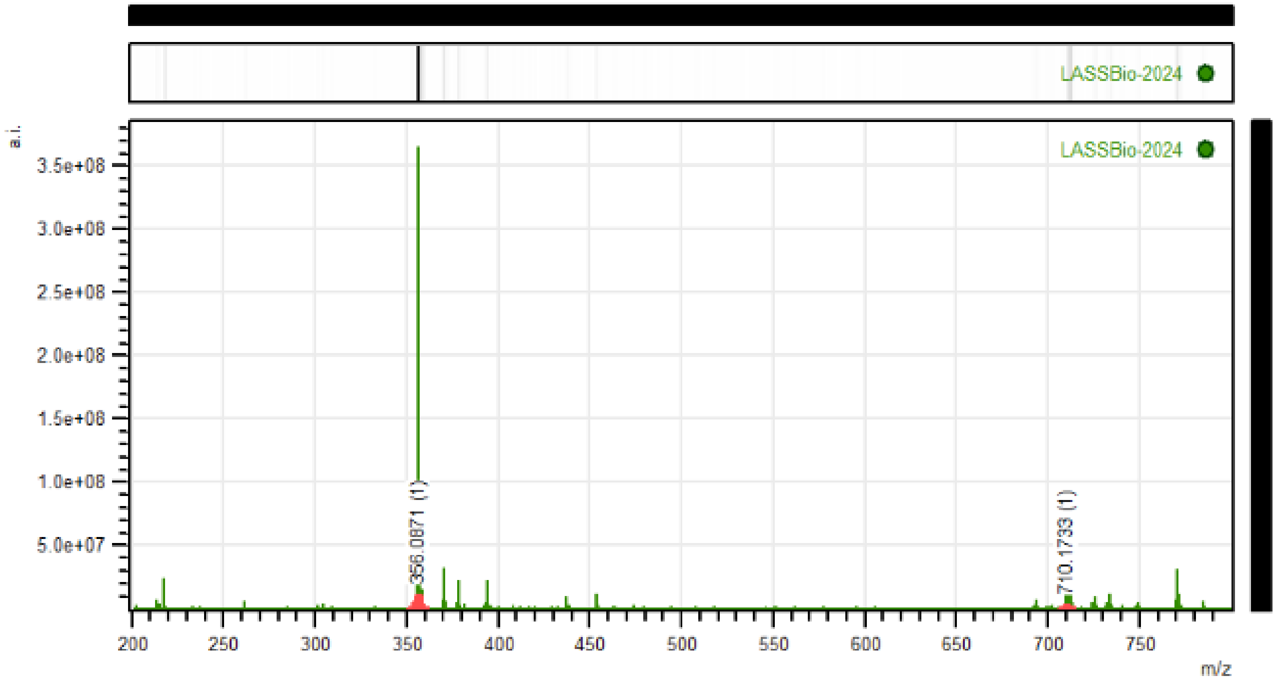

Figure 25 - HRMS of **5f** (ESI-FT-ICR) ($[M+H]^+$).

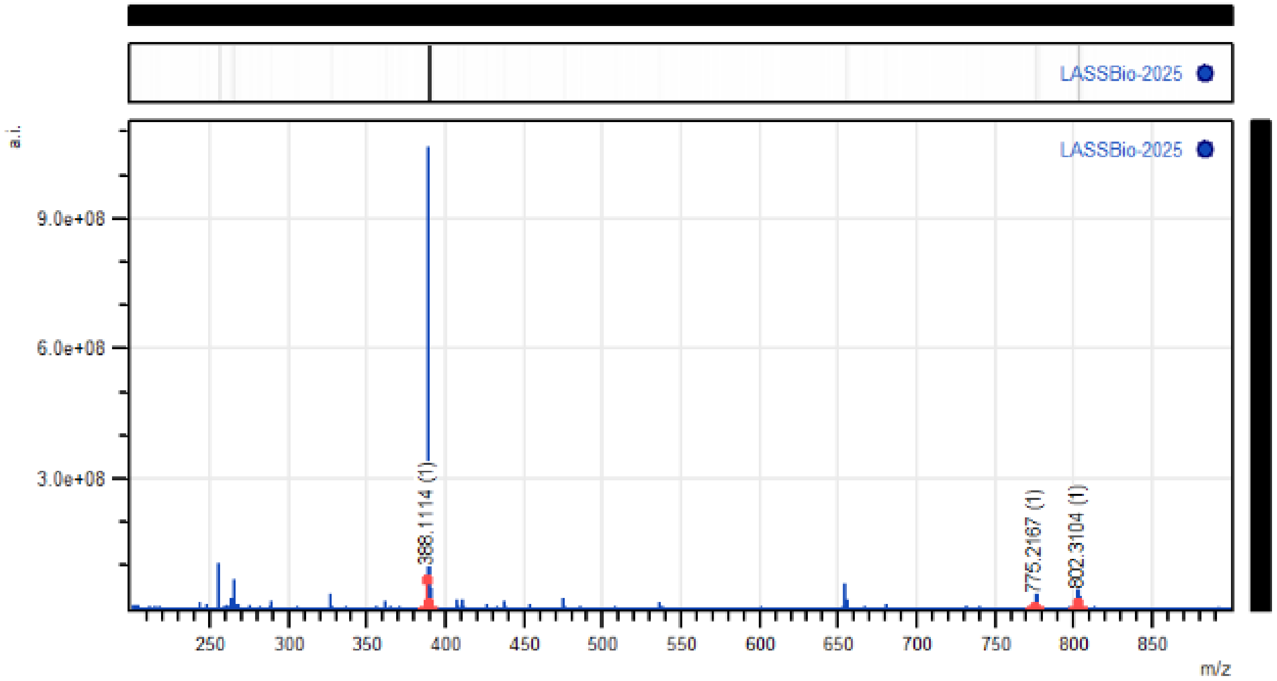

Figure 26 - HRMS of **5g** (ESI-FT-ICR) ($[M+H]^+$).

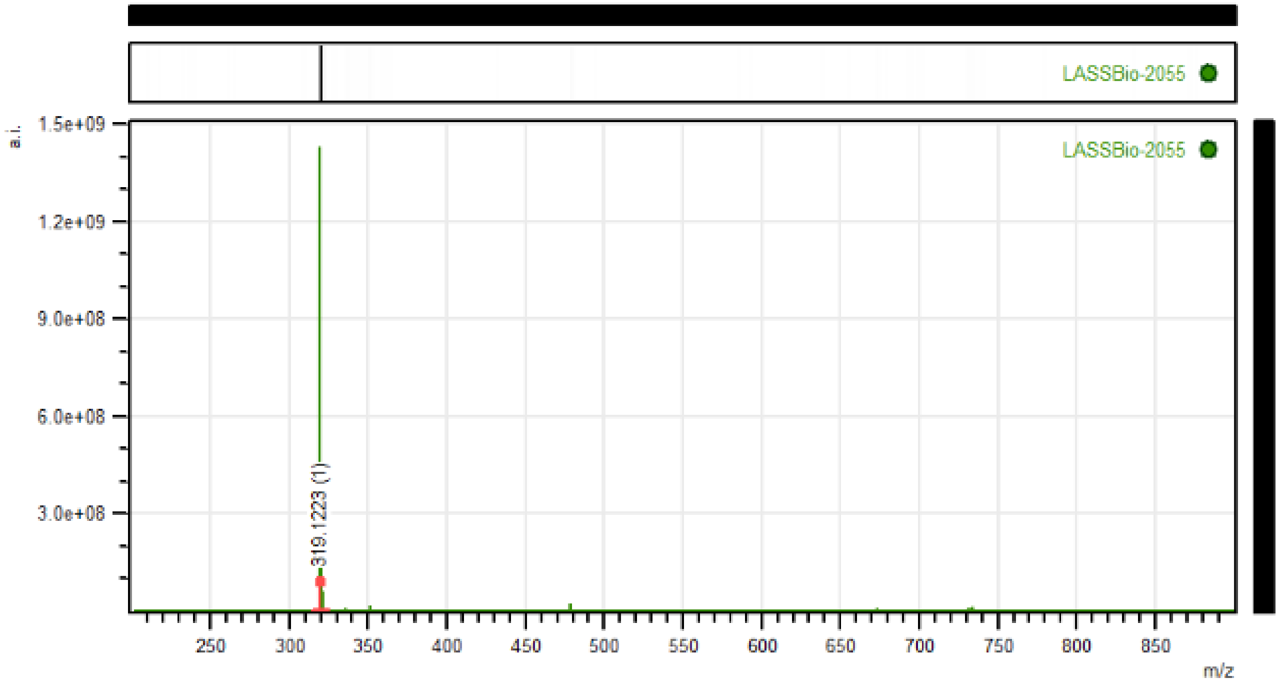

Figure 27 - HRMS of **5h** (ESI-FT-ICR) ($[M+H]^+$).

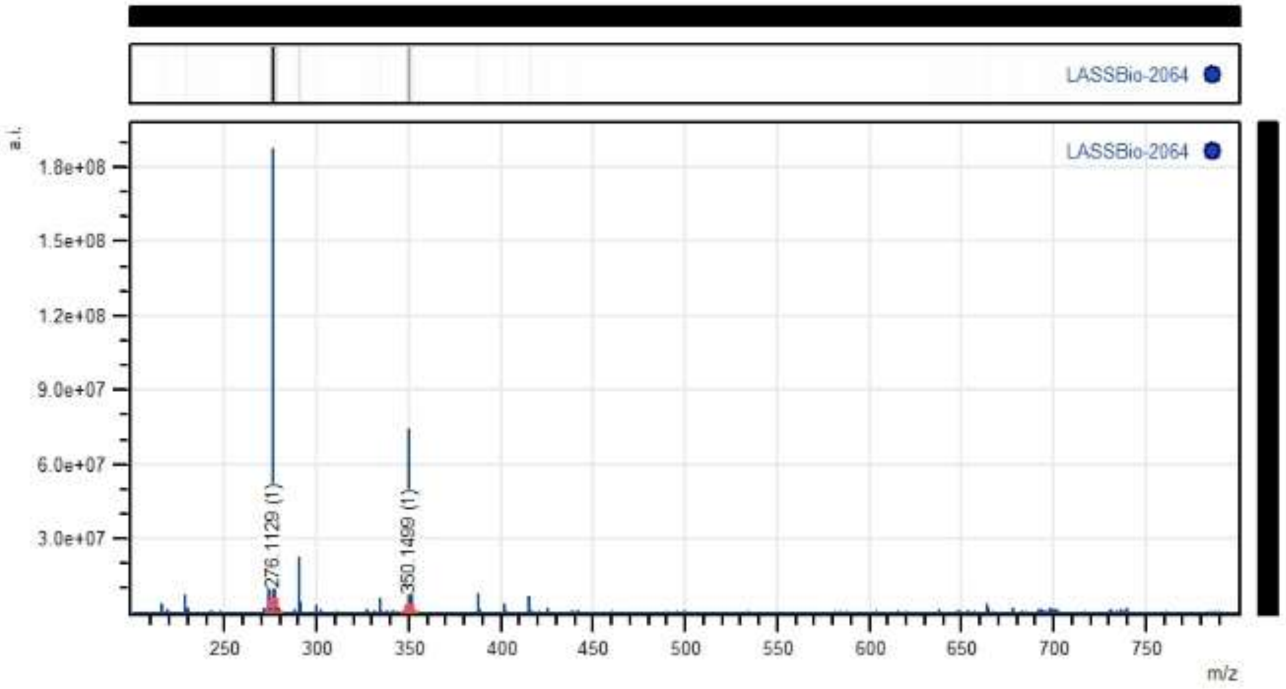

Figure 28 - HRMS of **10** (APCI-FT-ICR) ($[M+H]^+$).

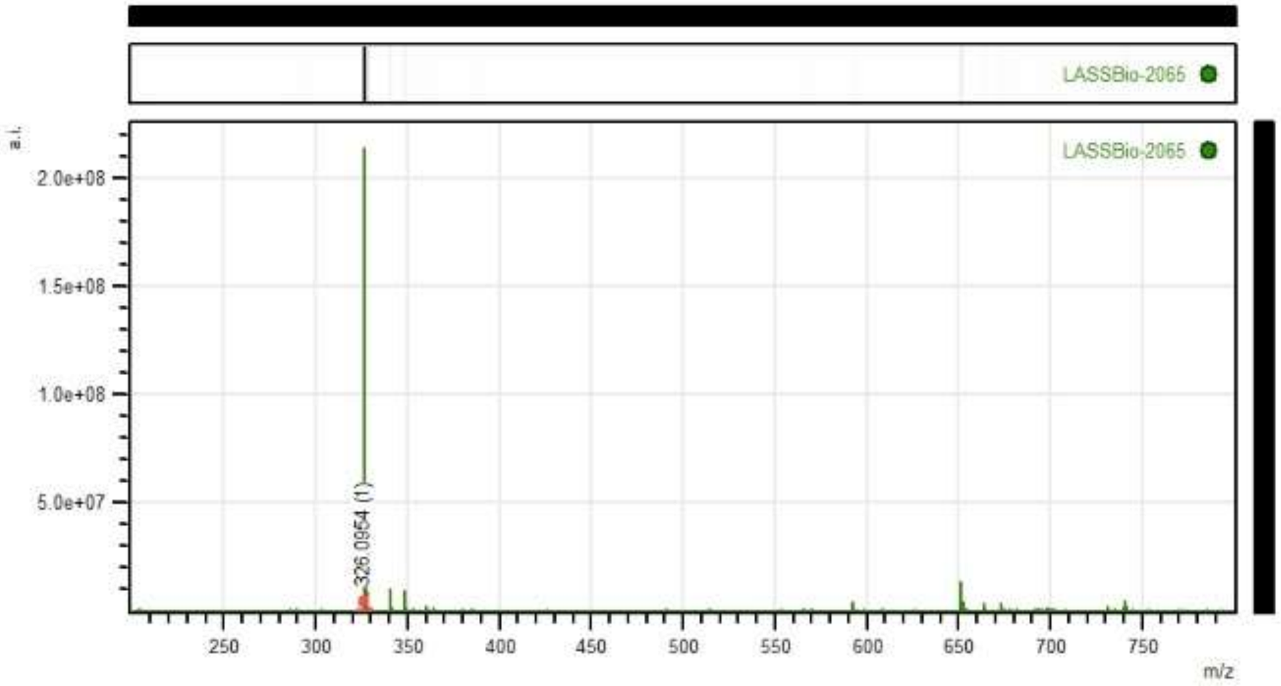

Figure 29 - HRMS of **11** (ESI-FT-ICR) ([M+H]$^+$).

Compound I.D.	Client Compound I.D.	IC_{50} (M)	nH	Test Concen-tration	% Inhibition of Control Values 1st	2nd	Mean	Flags 1st	2nd
ROCK1 *(h)*									
100040481-1	LASSBio-2020	1.3E-05 M	1.0	1.0E-06 M	19.9	14.3	17.1		
				3.0E-06 M	23.7	33.2	28.5		
				1.0E-05 M	43.5	47.0	45.2		
				3.0E-05 M	80.5	84.3	82.4		
				5.0E-05 M	70.0	76.3	73.1		

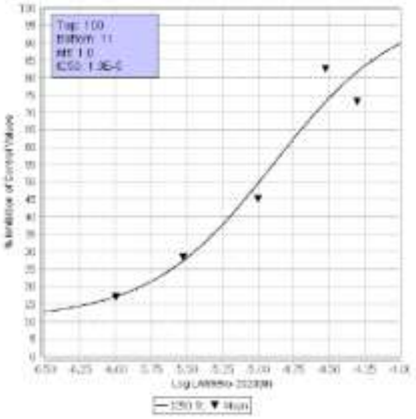

Figure 30 - Determination of IC_{50} of **5b** in human ROCK1.

Compound I.D.	Client Compound I.D.	IC_{50} (M)	nH	Test Concentration	1st	2nd	Mean	Flags 1st	2nd
ROCK2 *(h)*									
100040481-1	LASSBio-2020	7.1E-06 M	0.8	1.0E-06 M	13.6	13.5	13.6		
				3.0E-06 M	34.7	23.3	29.0		
				1.0E-05 M	51.1	59.4	55.3		
				3.0E-05 M	73.6	72.4	73.0		
				5.0E-05 M	57.1	29.3	43.2	{}, NSI	{}, NSI

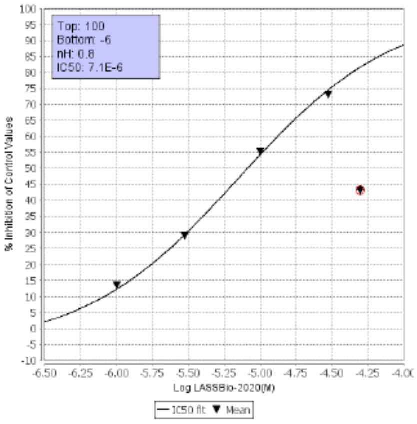

Figure 31 - Determination of IC_{50} of **5b** in human ROCK2.

Compound I.D.	Client Compound I.D.	IC_{50} (M)	nH	Test Concen-tration	% Inhibition of Control Values			Flags	
					1st	2nd	Mean	1st	2nd
100040481-3	LASSBio-2065	3.1E-06 M	0.9	1.0E-06 M	26.3	27.0	26.7		
				3.0E-06 M	54.8	44.4	49.6		
				1.0E-05 M	75.5	73.9	74.7		
				2.0E-05 M	80.6	88.5	84.5		
				3.0E-05 M	75.3	69.4	72.3	{}, NSI	{}, NSI

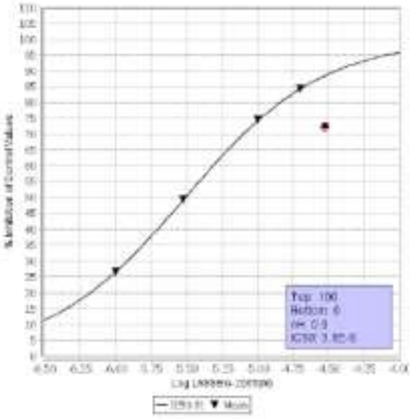

Figure 32 - Determination of IC_{50} of **11** in human ROCK1.

100040481-3	LASSBio-2065	3.8E-06 M	1.0	1.0E-06 M	31.7	3.9	17.8
				3.0E-06 M	36.0	53.9	45.0
				1.0E-05 M	76.9	79.0	77.9
				2.0E-05 M	86.5	81.1	83.8
				3.0E-05 M	81.9	77.8	79.8

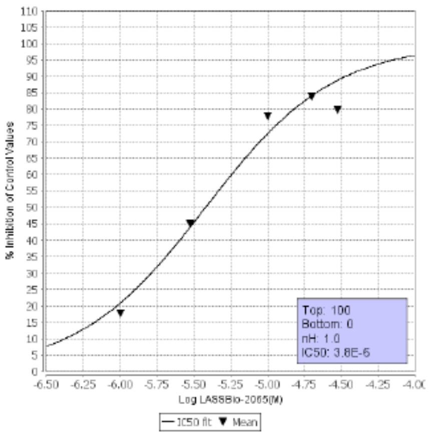

Figure 33 - Determination of IC_{50} of **11** in human ROCK2.

Compound I.D.	Client Compound I.D.	IC_{50} (M)	nH	Test Concen-tration	% Inhibition of Control Values			Flags	
					1st	2nd	Mean	1st	2nd
100040481-2	LASSBio-2064	N.C.	n/a	1.0E-06 M	-9.4	-10.8	-10.1		
				3.0E-06 M	-9.6	-7.7	-8.7		
				1.0E-05 M	-2.3	-1.5	-1.9		
				2.0E-05 M	6.1	4.0	5.1		
				3.0E-05 M	-1.9	-1.5	-1.7		

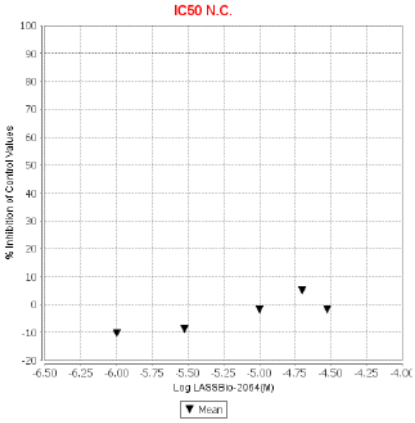

Figure 34 - Determination of IC_{50} of **10** in human ROCK2.

100040481-2	LASSBio-2064	N.C.	n/a	1.0E-06 M	-6.7	2.2	-2.2
				3.0E-06 M	2.2	0.1	1.1
				1.0E-05 M	-4.0	-4.0	-4.0
				2.0E-05 M	1.6	1.3	1.5
				3.0E-05 M	5.1	5.2	5.2

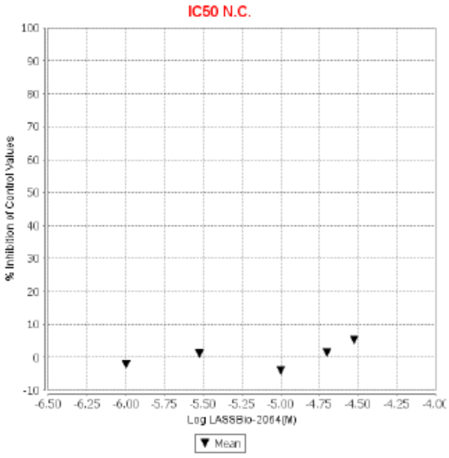

Figure 35 - Determination of IC_{50} of **10** in human ROCK1.

The final unit cell parameters, goodness-of-fit indicator and *R*-factors determined by a Rietveld refinement,(Figure 36) a = 11.4515(5) Å b = 13.2625(7) Å, c = 12.6577(6) Å, β = 116.191(4)º, V = 1725.02(16) Å^3, χ^2 = 2.088, R_{exp} = 2.106%, R_{wp} = 4.397% and R_{Bragg} = 1.171%.

Crystal data as well as details of the structure determination are shown inFigure 36 and Table 1.

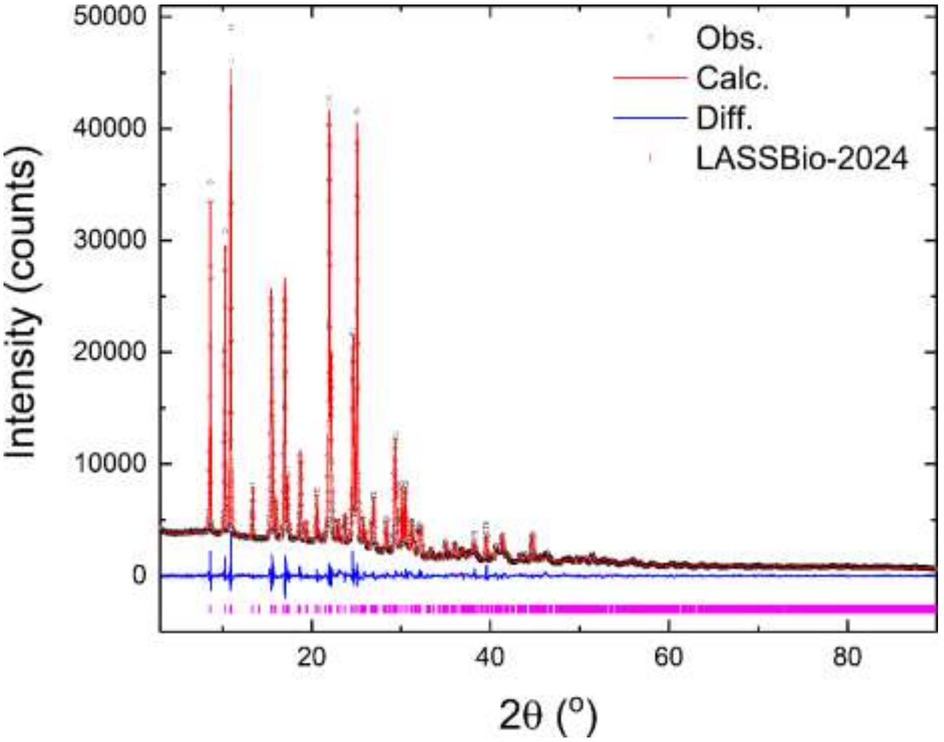

Figure 36 - Rietveld plot for **5f** (LASSBio-2024). The observed data is represented by the black open circles while the calculated pattern is indicated by the red line. The difference between the calculated and observed data is illustraded by the blue line at the bottom.

Chemical formula	$C_{16}H_{14}BN_3O_4S{\cdot}H_2O$
Formula weight (g mol^{-1})	373.19
Crystal system	Monoclinic
Space group	*P*2$_1$/*c* (Nr. 14)
a, *b*, *c* (Å)	11.4515(5), 13.2625(7), 12.6577(6)
β (°)	116.191(4)
Volume (Å^3)	1725.02(16)
Z, Z´	4, 1
ρ_{calc} (g cm^{-3})	1.43696(13)
T (K)	298
Data collection	
Diffractometer	STADI P
Monochromator	Ge(111)
Wavelength (Å)	1.54056
2θ range (°)	3-91.185
Step size (°)	1.05
Time per step (s)	200
Refinement	
Number of data points	5880
Number of contributing reflections	1462
Number of restraints	68
Number of refined parameters	129
R_p (%)	3.228
R_{exp} (%)	2.106
R_{wp} (%)	4.397
R_{Bragg}(%)	1.171
χ^2	2.088

Table 1 - Details from Rietveld refinement of the crystal structure of**5f**(LASSBio-2024).
